# Exploring Informal Caregivers’ Perception of the Olera Digital Caregiving Assistance Platform for Dementia Care: Mixed Methods Evaluation Study

**DOI:** 10.2196/92967

**Published:** 2026-07-03

**Authors:** Minh-Nguyet Hoang, Laura Kim, Louis Fisher, Logan DuBose, Marcia G Ory, Shinduk Lee, Tokunbo Falohun, Qiping Fan

**Affiliations:** 1 Naresh K. Vashisht College of Medicine, Texas A&M University, 8447 John Sharp Pkwy, Bryan, TX, 77807, United States, 1 214-985-7035; 2 Department of Public Health, College of Behavioral, Social, and Health Sciences, Clemson University, Clemson, SC, United States; 3 Department of Environmental and Occupational Health, School of Public Health, Texas A&M University, College Station, TX, United States; 4 Division of Health Systems and Community-Based Care, College of Nursing, University of Utah, Salt Lake City, UT, United States; 5 Department of Biomedical Engineering, College of Engineering, Texas A&M University, College Station, TX, United States

**Keywords:** dementia, Alzheimer disease, family caregiving, caregiving challenges, Technology Acceptance Model, artificial intelligence, usability, ease of use

## Abstract

**Background:**

Informal caregivers of people living with dementia often experience high rates of caregiver burnout while providing care. Although there are many websites and mobile apps available to help caregivers, many do not use digital tools. The Olera platform was developed to be an easily adoptable web-based support tool, connecting caregivers with long-term services and supports, financial assistance, and educational resources. The platform was developed based on the Build-Measure-Learn framework with input from caregiver needs assessments and usability studies.

**Objective:**

This study aims to evaluate the quantitative and qualitative feedback of informal caregivers of people living with dementia on the second iteration of the Olera platform. The primary objective was to assess caregivers’ acceptance of this caregiving platform. The secondary objective was to use qualitative methods to explore (1) the study cohort’s challenges in daily caregiving to determine and compare them with prior literature, (2) their experience when using the Olera platform, and (3) their attitudes toward integrating artificial intelligence in caregiver services for future studies and platform development.

**Methods:**

Caregivers were recruited through various sources and screened for eligibility through an initial survey. Participants used the platform for 4 weeks and completed a survey with an adapted Technology Acceptance Survey (TAS) and qualitative open-ended questions at the end of the testing period. TAS responses were summarized with descriptive statistics, while ANOVAs, *t* tests, and linear regressions were used to compare the differences in the overall TAS scores by caregiver characteristics. Qualitative feedback data on the platform’s usefulness were analyzed via a thematic analysis framework approach.

**Results:**

A total of 65 caregivers in the United States completed the study, with a mean age of 59.9 (SD 9.8) years. The majority were female (61/65, 95.3%), non-Hispanic or Latino White (45/65, 69.2%), and the adult child of their care recipient (42/65, 64.6%). Evaluation of the Olera platform showed a high acceptance rate, with each TAS item scoring above 5.0 and an overall TAS score of 5.83 (SD 0.85) out of 7. Higher platform use frequency was associated with higher TAS ratings in technology acceptance (*F*_3,61_=7.88, *P*<.001). Thematic analyses elicited the caregiving challenges, evaluation of the Olera platform, and feedback on artificial intelligence–assisted support.

**Conclusions:**

The Olera platform is an example of a beneficial web-based tool, though key features were requested to be included in the next iteration. Additionally, data supported prior findings regarding informal caregiver challenges and the insufficiency of conventional support mechanisms, indicating a need for more innovative digital solutions. Future research and development efforts using the Build-Measure-Learn approach are necessary to further iterate the platform’s key features, enhance the tool, involve more informal caregivers in its improvements, and serve as a model for customizable, person-centered online care support.

## Introduction

### Background

Dementia is a collective term for a group of symptoms often manifesting as a significant decline in cognitive, behavioral, and/or functional abilities that interfere with an individual’s ability to perform activities of daily living (ADLs) [1,2]. Alzheimer disease (AD) is the most common cause of dementia, accounting for an estimated 60%‐80% of dementia cases [2,3]. With yearly increases in aging populations, the number of individuals with AD and AD-related dementia is expected to grow, creating further caregiving demands to support their ADLs [2].

Family caregivers of people living with dementia play an integral role in the safety and health of people living with dementia, accounting for an estimated 19.2 billion hours of unpaid care in 2024 [2]. Depending on the stage of dementia, the demands for caregiving of people living with dementia may vary from minimal help with their ADLs to intensive, full-time care [2,4]. Although many informal caregivers report a sense of satisfaction from caring for their loved ones, they are often at greater risk of stress, depression, and anxiety [5-7]. Additionally, caregivers of people living with dementia often balance multiple responsibilities and face financial, legal, and functional challenges during their caregiving journey [8]. Their caregiving challenges are compounded by a general lack of support, accessible information, and resources available to caregivers and their loved ones with dementia [2,8-11]. Despite relative increases in the number of people living with dementia in Hispanic or Latino and African American populations compared to non-Hispanic or Latino White populations [2,11,12], there is a decrease in diagnosis of dementia and use of caregiving services in ethnic minority groups, potentially due to a multitude of barriers, including a lack of culturally appropriate care and negative perception of mainstream services [12,13].

While various support services and caregiving tools exist, such as psychosocial interventions, web-based psychoeducation programs, and mobile apps, many do not provide caregivers with individualized and relevant information necessary for their care recipient’s needs [14-17]. Caregivers desire customizability, person-centered, and creative design solutions in their caregiver technology solutions [17-23]. Artificial intelligence (AI)–driven technology solutions for informal caregivers can readily provide tailored, precise information and services that enhance the support system for these individuals [20-22,24]. Despite these potential benefits, most studies on AI technology solutions for informal caregivers have not involved them as users in the development and testing stages [25]. While Allahvirdi et al [19] reported an overall willingness to use AI caregiver tools in a small cohort of informal caregivers, AI hesitancy persists due to concerns about data security and the quality of data, particularly in the lack of transparency in data sources and in medical settings [26-29]. To the authors’ knowledge, willingness to adopt interactive AI apps is not well documented within larger cohorts of informal caregivers.

The theory of planned behavior suggests that the more favorably an individual perceives a technological intervention, the higher the likelihood of its adoption [30]. Several frameworks are commonly used to evaluate users’ overall attitude toward technological interventions, including the System Usability Scale (SUS), Usability Metric for User Experience, and Technology Acceptance Model (TAM) [31-33]. Of these, the TAM and its variations—extending to constructs that include subjective norms, self-efficacy, and other external factors—are comprehensive and popular as a framework to assess novel technologies [34,35]. Using the theoretical framework of the TAM, the Technology Acceptance Survey (TAS) is designed and adapted according to relevant features of a technology to evaluate the user’s behavioral intention to adopt such technology. It is important to note that within the literature, behavioral intention and acceptance are commonly used interchangeably [36]. We follow this convention and use acceptance when referring to behavioral intention.

While necessary to distinguish actual end use and acceptance, the former is a reliable predictor of the latter, and, considering the difficulties in assessing actual use in research settings, acceptance is a desirable surrogate [36]. Furthermore, according to the TAM, perceived usefulness and perceived ease of use are the 2 most important factors in predicting technology acceptance and, thus, are important in determining the likelihood of an individual’s adoption or end use of a technology [35]. Perceived usefulness is the extent to which a person believes technology enhances job performance, and perceived ease of use is the extent to which a person believes the technology used is effortless [37].

A user’s perceived usefulness and perceived ease of use are influenced by a technology’s functionality and usability [37]. While usability describes how effective and comfortable a technology is, functionality refers to the specific features, operations, and designs that enable a technology to help users meet their goals [38-40]. While distinct, a technology’s usability affects an individual’s perception of its overall functionality: a technology with a poor interface and usability can lower the perceived usefulness and ease of use due to increased difficulty in navigating the product [40]. Moreover, an individual’s digital literacy (awareness to properly function in digital environments), along with their cognitive and social ability [41] and linguistic literacy (ability to consciously access and view a language from various perspectives or modalities [42,43]), have been shown to influence these perceptions [41,44,45]. Similarly, health literacy, which is the ability to obtain and apply knowledge related to one’s health care needs [46], particularly electronic health literacy, has influenced the adoption of health-related technologies [47,48]. Users with higher literacy levels often report greater usability and functionality, and, thus, a higher acceptance of a novel technology, as reflected in higher TAM scores in previous studies [41,49]. In relation to this, the rate of adoption of a technology refers to the speed at which a new technology will begin to be used after it is introduced [50]. This rate can be influenced by factors such as the perceived complexity of a technology and its compatibility with existing practices and technologies [50].

Based on the theory of planned behavior, we evaluated a web-based platform (Olera) in understanding caregivers’ needs and the rating of the platform in a preliminary usability study among a small cohort of caregivers, with high technology ratings across engagement, functionality, aesthetics, and information quality [8,17]. The Olera platform is an example of a technology that provides customizable, person-centered recommendations on education and resources for long-term services and support and for caregivers of people living with dementia to address their financial, functional, and legal barriers to caregiving. Based on preliminary feedback [8,17,18], the website was further refined using the Build-Measure-Learn framework of user-centric software development to better address caregivers’ needs at their care recipient’s stage of dementia [51].

### Objectives

The overarching goal of the study is to investigate informal caregivers’ perceptions of the Olera platform in order to continue iterative refinement of the platform. Our major study aim is to evaluate the acceptance of the second iteration of the Olera platform using an adapted TAS. We secondarily aim to explore (1) this study cohort’s challenges in daily caregiving and compare them with prior literature, (2) their subjective experience when using the Olera platform, and (3) their attitudes toward integrating AI for assistance in caregiver services via open-ended questions for future studies and platform development.

## Methods

### Overview

Participants in the study engaged with the Olera platform for 4 weeks. Data on participants’ characteristics, platform usability, and likelihood of adopting a technology were collected using an initial eligibility survey and an exit survey at the conclusion of the 4 weeks. The exit survey contained open-ended questions to collect feedback on platform features to inform future iterations of the platform and a modified TAS reflecting the TAM. The TAS used a 7-point Likert scale to assess the perceived usefulness and ease of use of the platform among participants.

### Platform Development

The Build-Measure-Learn cycle of lean product development guided our framework for creating and improving the Olera platform [51]. This approach emphasizes the rapid prototyping of new features and the structured evaluation of user-centered metrics to inform the iterative refinement of the platform’s user interface and user experience and content [33,36,52,53]. It was first used through an initial needs assessment of informal caregivers completed in Phase I of this project [8], followed by the initial prototyping phase, where the platform received a 4.57/5 on the Mobile Application Rating Scale with a small cohort of informal caregivers in Texas [17]. The platform’s core functionalities were initially shaped by the needs assessment and included a comprehensive service provider directory; educational guides covering legal, financial, and estate planning decisions; and a personalized recommendation system that uses an algorithm to curate provider listings and caregiving resources tailored to each caregiver’s unique situation. The platform also featured the Olera Score, a composite rating from publicly available data that integrates measures of provider reputation, value, and information availability to help families compare care options. By synthesizing these dimensions into a single standardized score, the Olera Score was designed to facilitate systematic comparisons of care options and reduce information asymmetry for caregivers navigating complex care decisions.

After initial usability testing with informal caregivers, their feedback informed the Phase II build, which expanded on the initial prototypes, including a refined webpage layout, updated content, and new features, such as a caregiver discussion board and expanded provider listings across assisted living, memory care, nursing homes, home health, and personal care. Additionally, service provider browsing and filtering search tools were improved, and multichannel touchpoints, such as automated communications of key tips and resources through text messages and emails, were used to increase engagement.

Regarding the platform layout, the Phase II platform was optimized with a minimalist design, including only 1‐3 call-to-action buttons per page to avoid decision paralysis when navigating the platform, consistent color schemes, and a large font with high-contrast text. To allow users to effortlessly scan the webpages and focus on key call-to-action buttons, visual clutter, including extensive instructions, was minimized. Recommended providers and resources were also added to assist users in finding resources related to their search queries. The user experience design (eg, how users navigate from one page to another) prioritized fast page loading, avoidance of broken links, and consistent arrangement of navigation elements across subpages.

Additionally, informed by feedback from Phase I user testing, the various topics and services covered on the Olera platform were curated more intentionally to align with the established stages of caregiving, thereby appealing to informal caregivers at different points in the caregiving process [54]. Content was developed by authors with domain expertise in memory care, senior care coordination, long-term services and supports, and clinical medicine. The content was written at an elementary grade reading level, featuring 100‐ to 500-word pieces for a streamlined and easy reading experience. When users created an account, they were prompted to answer a personalization quiz for searching and filtering by service type (eg, home health, memory care, and legal aid), geography (eg, state and ZIP code), and topic category to further individualize their experience and to receive recommendations on service providers, resources, and learning materials such as articles and videos (Multimedia Appendix 1).

To increase engagement with the platform during the study, participants received text messages every 2‐3 days with links to educational articles on the platform or instructions to interact with it. They also received weekly emails summarizing all the recommended links sent via text message. While enrollment in the email communications was required, opting into the text message sequences was optional. This was an automated process with open rates and link clicks monitored periodically to ensure frequent touchpoints with participants. The emails and text messages were modified to encourage engagement and use of different aspects of the web-based platform in conjunction with any design or product updates, which were minimal, made to the Olera platform (Multimedia Appendix 2)

### Participant Recruitment and Eligibility

Participants were recruited and completed the study from October 2023 to July 2025 across the United States. Participants were recruited primarily through 2 outlets: previous study participation [8,17] or Facebook (Meta Platforms) advertisements. Previous study participants were individuals who completed prior studies during which they were exposed to the first version of the Olera platform for 1 interview session without extensive use of the website [8,17]. Given this minimal exposure to the platform and subsequent iterations of the platform based on participant feedback, previous study engagement was not deemed a confounding variable or an exclusion criterion for this study. Facebook advertisements contained forms that collected contact information from potential participants. Interested individuals were followed up with a series of up to 4 phone calls by research personnel to provide information regarding the study and complete the initial eligibility survey during the call. After the initial recruitment call, research leads were also enrolled in automated email and text sequences containing online links to the eligibility survey as an additional touchpoint to recruit study participants (Multimedia Appendix 3).

The eligibility survey collected the relevant information to examine whether an interested individual meets the following inclusion criteria: (1) is 18 years and older; (2) is a nonpaid caregiver of people living with dementia; (3) is the adult child, spouse or partner, other family member, or legal guardian of people living with dementia; (4) is engaged in making legal, financial, senior living, or medical decisions for people living with dementia; and (5) has access to a smartphone or computer with internet access.

Following completion of the initial eligibility screening survey, qualified participants were enrolled in the study to use the Olera platform for a subsequent 4 weeks. Full enrollment criteria consisted of (1) completion of the eligibility survey and meeting the inclusion criteria, (2) creation of an account on the Olera platform, and (3) enrollment in email communications during the study. The study aimed to recruit approximately 150 participants initially, with the anticipation of 50% completing the full 4-week study and exit survey that included a modified TAS to meet the minimum requirement for bivariate analyses [55]. During the onboarding process, caregivers were also given a quick orientation of the digital platform via phone call.

### Ethical Considerations

Ethical approval was obtained from the Texas A&M University Institutional Review Board (number 2021-0943D). The study personnel asked all participants for electronic informed consent in the eligibility screening survey, either with the assistance of study personnel or independently. Upon completion of the initial eligibility survey and determination of meeting the inclusion criteria for the study, participants were presented with the informed consent document. The informed consent document provided instructions on how to convey their consent to participate in the study and their willingness to be followed up via the survey platform. The consent form covered important information, including the rationale for inclusion, the research objectives, the voluntariness of participation with the option to withdraw at any point, the anticipated participation duration and procedures, potential risks, benefits, and costs of participation, and how their confidentiality will be protected.

### Data Collection

A concurrent mixed methods data collection was used in this study that consisted of quantitative (responses to closed-ended questions) and qualitative data (responses to open-ended questions) collected via 2 surveys at the beginning and at the end of the 4-week study: an initial eligibility survey and an exit survey (including the TAS). Quantitative and qualitative data were integrated in the analysis and interpretation phase. Specifically, the quantitative TAS and the open-ended questions for user experience and platform usefulness were integrated for evaluation of the platform acceptance. Open-ended question topics included caregiving challenges, subjective user experience of the Olera platform, and AI use in caregiving technology. During the study period, minor refinements were implemented in the Olera platform that did not significantly alter the user experience or require additions to the exit survey. While no additional features were evaluated, in January 2025, the exit survey was expanded to include questions on potential platform features and a few more detailed open-ended questions to gather participants’ feedback on AI-assisted caregiving support.

### Assessments and Measurements

#### Primary Outcome: Technology Acceptability

The primary quantitative outcome was caregiver-rated acceptability of the Olera platform, assessed at the end of the 4-week study using an adapted TAS based on the TAM [34-37]. The TAS is a widely accepted standardized questionnaire to assess a user’s perceived usefulness and perceived ease of use of a digital tool [33,36], and was adapted to reflect the specific functions and interface of the Olera platform, with item wording tailored to assess the perceived usefulness and perceived ease of use of the Olera platform (Multimedia Appendix 4) [33,36,37]. The adapted TAS consisted of 29 items, with a total possible score of 203. Participants provided ratings for each TAM item using a 7-point Likert scale, with higher values reflecting positive perceptions of a technology. Item-level and the overall TAS score were used to describe the acceptability of the platform in the sample. An overall TAS score of 5.0 or higher was interpreted as indicating generally favorable perceptions of the platform. This benchmark was used for descriptive interpretation only, based on prior literature using similar TAS measures and guidance on Likert scale interpretation [56].

#### Secondary Descriptive Measures: Platform Use and User Feedback

Participants also reported their engagement with the Olera platform during the 4-week study, including frequency of use per week and total time in minutes spent using the website. In addition, the exit survey included open-ended questions about participants’ experience using the platform, aspects they found helpful, and recommendations for improvement. These responses were used to triangulate the quantitative acceptability findings and to inform future platform refinement.

#### AI-Related Questions

To understand the willingness and barriers to the use of AI for caregivers for future platform features, we asked participants if they were willing to use AI-assisted caregiver support, with response options of either “yes” or “no.” Given hesitancy in adopting AI as a tool [26-29], open-ended questions were also asked about their barriers to using AI-assisted caregiving support and factors that would make them more confident in using such technology. The responses were coded using thematic analysis to identify common themes regarding barriers and motivators for AI adoption among caregivers for future platform development.

#### Participant Characteristics and Caregiving Context

We collected the participant background characteristics and caregiving characteristics to understand whether the major platform evaluation outcomes differed by the background characteristics of the participants. Open-ended questions about participants’ caregiving challenges were also asked to explore the challenges of the study cohort [1,8]. The caregiver characteristics collected included age, sex, race, ethnicity, the highest level of education completed, employment status, general financial status, caregiving role, length of providing care, literacy in English, willingness to adopt a new technology, digital literacy on a 5-point Likert scale, and health literacy via questions adapted from the Brief Health Literacy Screening Tool [57]. Caregivers were also asked to self-identify the rate at which they would adopt a new technology to determine whether it affected their overall perception of the Olera platform, including their TAS scores. The options to self-identify their likely rate of adoption of a new technology were as follows: when it becomes popular, after most peers, before most peers, and one of the first to try. Caregivers also reported their care recipient’s subjective stage of dementia based on the Functional Assessment Staging Tool [58].

### Data Analysis

For the quantitative survey data collected, descriptive statistics were used to summarize caregiver characteristics, TAS responses, and platform engagement. Continuous variables were summarized using means and SDs, and categorical variables were summarized using frequencies and percentages. Differences between participants who completed the exit survey and those lost to follow-up were examined descriptively. Independent-samples *t* tests, Fisher exact tests, and chi-square tests were conducted comparing the caregivers’ characteristics between 2 groups.

The primary quantitative outcome was the overall TAS score at the end of the 4-week study. For each participant, the overall TAS score was calculated as the mean of all TAS items. Positively worded items (7=strongly agree, 6=agree, 5=somewhat agree, 4=neutral, 3=somewhat disagree, 2=disagree, and 1=strongly disagree), and negatively worded items (7=strongly disagree, 6=disagree, 5=somewhat disagree, 4=neutral, 3=somewhat agree, 2=agree, and 1=strongly agree) were sorted so that higher values consistently reflected more favorable perceptions of the platform. Item-level means and SDs and the overall TAS mean with 95% CI were used to describe caregiver-rated acceptability of the Olera platform.

Exploratory analyses were conducted to examine whether TAS scores varied by caregiver characteristics. Group comparisons were conducted only for categories with an adequate sample size for meaningful interpretation. A 1-sample *t* test was conducted to compare the overall mean TAS score with the study’s chosen threshold of 5.0 based on the literature [56]. Bivariate analyses were performed to examine differences in TAS scores by caregiver characteristics. Specifically, 1-way ANOVAs were conducted to examine differences in TAS score across caregiver characteristics with more than 2 categories, while independent-samples *t* tests were used for caregiver characteristics with 2 groups. Linear regression was conducted for continuous caregiver variables, such as age and time spent interacting with the platform. All analyses were conducted using Stata (version 17.0; StataCorp).

For the qualitative data collected through open-ended questions, the qualitative research framework approach with thematic analysis was conducted to identify and organize themes and study findings [59]. This framework approach is widely used in analyzing qualitative data in health research since it was developed in the 1980s [59,60]. First, the researchers (MNH, QF, and LD) developed a preliminary codebook using the survey questions, prevalent caregiving challenges, and several randomly selected TAS responses to develop the initial themes. The researchers then selected several more survey responses to code together and finalized the codebook in a group meeting (Multimedia Appendix 5). Finally, 2 coders (MNH and QF) independently coded all the transcripts using an Apple version of the NVivo (version 15.2.1; Lumivero) software. The intercoder reliability was evaluated using intercoder agreement [53]. The intercoder agreement rate was 94.52%, indicating that the intercoder reliability was excellent. The study personnel (MNH, QF, and LD) discussed any disagreements and reached a consensus after thorough review. After the coding process, the researchers identified major themes with subthemes nested under each major one, charting these findings in a Microsoft Excel sheet (Microsoft Corp; Multimedia Appendix 5.

## Results

### Participants’ Characteristics

**Figure 1.**
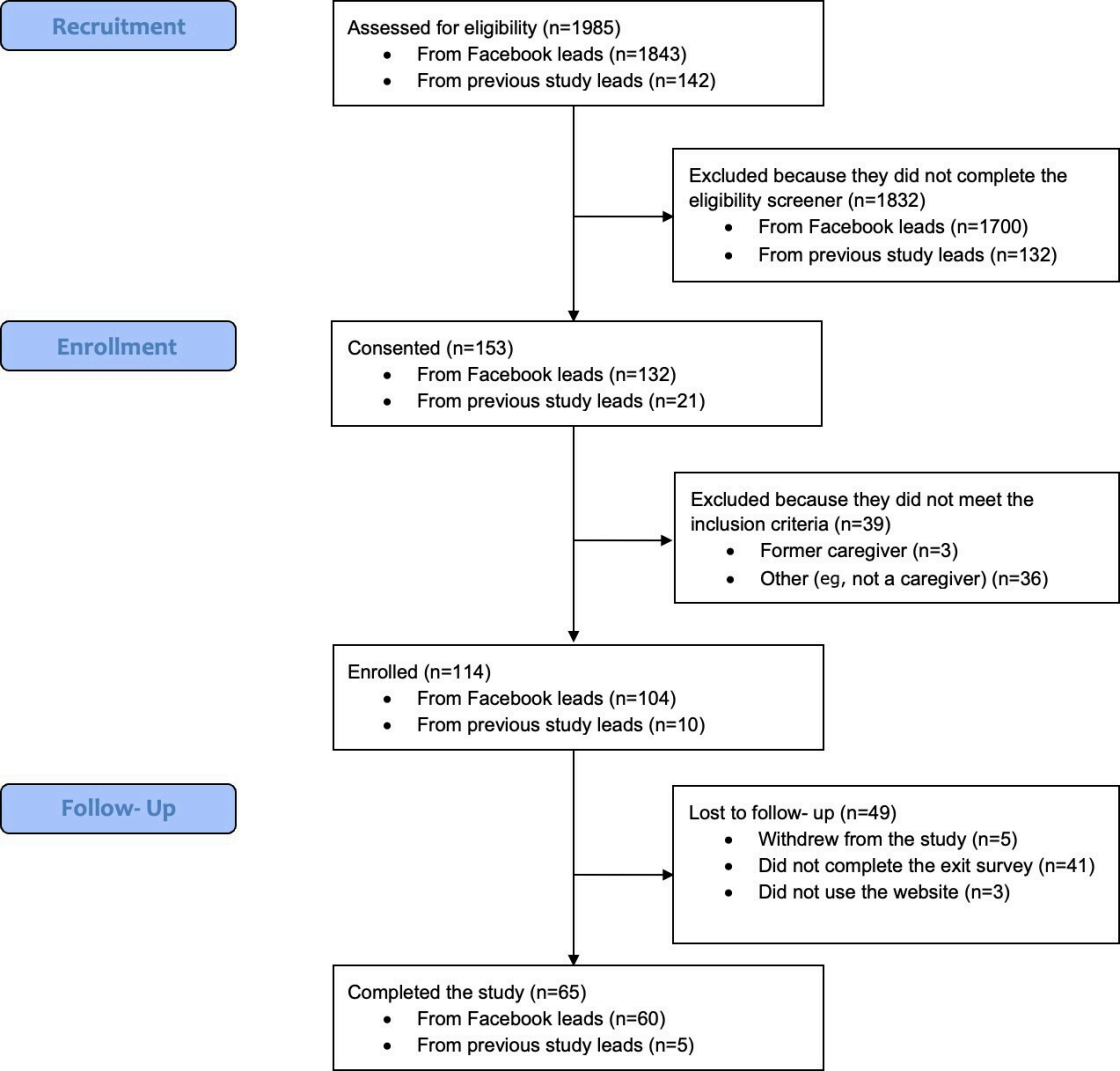
CONSORT (Consolidated Standards of Reporting Trials) diagram of recruitment, enrollment, and follow-up.

Out of the initial 142 previous study participants and 1843 Facebook respondents, 153 (153/1985, 7.71%) completed the initial eligibility survey and met the eligibility criteria. Among the eligible individuals, 74.5% (114/153) fully enrolled in the study by creating an Olera account and joining the email list, and 57% (65/114) completed the technology acceptance survey. Of the 65 participants, 42 completed the study after the Olera platform and exit survey were updated. The process of recruitment, enrollment, and follow-up is presented in the CONSORT (Consolidated Standards of Reporting Trials) diagram (Figure 1).

Characteristics of participants who completed the study (N=65) are summarized and described in Table 1 and Multimedia Appendix 6. The mean age was 59.9 (SD 9.8) years. Most were female (61/65, 93.8%) and had at least an associate’s degree (46/65, 70.8%). The majority of the 65 individuals identified as non-Hispanic White (45/65, 69.2%), and the remaining represented non-Hispanic Black or African American, Hispanic or Latino, Asian, Native American or Alaskan Native, or multiracial groups. The average household size was 2.5 (SD 1.0) individuals. Respondents reported intensive care experience, spending an average of 5.6 (SD 9.8) years as a caregiver and 83.6 hours weekly caregiving. In terms of relationship status, the majority of participants were married (37/65, 56.9%), followed by widowed, divorced, or separated (14/65, 21.5%), or never married (12/65, 18.5%). Regarding work status, the majority of participants (39/65, 60%) were not currently employed, though 21.5% (14/65) worked full-time and 16.9% (11/65) worked part-time. Financially, 24.6% (16/65) of participants reported a total household income below US $50,000, 60% (39/65) reported a total household income between US $50,000 and US $100,000, and 13.8% (9/65) reported a total household income above US $100,000. Most respondents reported being the adult child of the care recipient (42/65, 64.6%), with the second most common caregiver relationship being spousal (16/65, 24.6%). In terms of caregiving responsibilities, the vast majority were involved in living arrangements (143/153, 93.3%), medical decision-making (149/153, 97.4%), legal or financial tasks (144/153, 94.1%), and 69.3% (106/153) were looking for caregiving services.

**Table 1.** Characteristics of participants who completed the 4-week study (N=65).

Variables	Value
Demographics and caregiving characteristics	
Age (years), mean (SD)	59.9 (9.8)
Sex, n (%)	
Female	61 (93.8)
Male	3 (4.7)
Did not respond	1 (1.5)
Race or ethnicity, n (%)	
White or Caucasian	45 (69.2)
Black or African American	9 (13.8)
Hispanic or Latin American	6 (9.2)
Other (Asian, Native American or Alaskan Native, and Multiracial)	5 (7.7)
Education, n (%)	
Graduate degree	16 (24.6)
Bachelor’s degree	21 (32.3)
Associate degree	9 (13.8)
Some college, but no degree	14 (21.5)
High school degree or equivalent	4 (6.2)
Did not respond	1 (1.5)
Employment status, n (%)	
Retired	25 (38.5)
Full-time employed	14 (21.5)
Part-time employed	11 (16.9)
Not employed, not looking for work	11 (16.9)
Not employed, looking for work	2 (3.1)
Disabled, unable to work	1 (1.5)
Did not respond	1 (1.5)
Relationship with care recipients, n (%)	
Adult child	42 (64.6)
Family member	6 (9.2)
Legal guardian	1 (1.5)
Spouse or partner	16 (24.6)
Technology proficiency (out of 5), mean (SD)	3.9 (1.0)
Digital and media literacy (out of 5), mean (SD)	3.9 (0.9)
Adoption of new technology, n (%)	
When it becomes popular	24 (36.9)
After most peers	17 (26.1)
Before most peers	15 (23.1)
One of the first to try	9 (13.9)
Olera platform interaction frequency, n (%)	
Daily	10 (15.4)
4‐6 times per week	18 (27.7)
2‐3 times per week	20 (30.8)
Once per week	17 (26.1)
Olera platform use (in minutes over past 4 weeks), mean (SD)	187.0 (167.0)

In terms of digital literacy and technology use, there was an average technology proficiency of 3.9/5 (SD 1.0) and a digital and media literacy average of 3.9/5 (SD 0.9). When asked how quickly respondents would adopt a new technology, 13.9% (9/65) selected being one of the first to try, 23.1% (15/65) selected before most peers, 36.9% (24/65) selected when it becomes popular, and 26.1% (17/65) after most peers. A high level of general and health literacy was noted, with all participants reporting an ability to write a personal letter in English and being able to easily follow medical instructions. Moreover, 98.5% (64/65) reported having the ability to confidently complete medical forms. However, it should be noted that some participants struggled, navigating some health care aspects, such as reporting difficulty understanding written health information (2/65, 3.1%), prescription labels (3/65, 4.6%), or health care provider explanations (3/65, 4.6%).

We compared baseline characteristics of participants who completed the exit survey with those lost to follow-up. No major differences were observed in measured baseline characteristics, although participants lost to follow-up reported somewhat higher caregiving hours per week; these comparisons are provided in Multimedia Appendix 7.

### Participants’ Platform Usage

During the 4-week study, 15.4% (10/65) of respondents reported using the Olera platform daily, 27.7% (19/65) reported using it 4‐6 times weekly, 30.8% (20/65) reported using it 2‐3 times weekly, and 26.1% (17/65) reported using it once weekly. Respondents used the platform for an average of 187.0 (SD 167.0) total minutes during their participation in the study.

### Technology Acceptance

A total of 65 participants completed the exit survey that included the modified TAS to evaluate the perceived usefulness and ease of use of the Olera platform. The means and SDs for each survey item were reported along with the overall average among all items (Multimedia Appendix 8). Participants reported an overall mean TAS score of 5.83 (SD 0.85; 95% CI 5.62‐6.04). Item-level mean TAS scores ranged from 5.31 (SD 1.38) to 6.37 (SD 0.72), showing generally favorable ratings across items (Multimedia Appendix 8). Response distributions were similarly skewed toward favorable ratings, and low item scores (<4) were uncommon (Multimedia Appendix 9).

Exploratory analyses examined the differences in overall TAS scores by platform engagement (Table 2; Multimedia Appendix 6). Higher platform interaction frequency was associated with greater technology acceptance (ANOVA *F*_3,61_=7.88, *P*<.001). Participants who used the platform daily reported the highest TAS scores (mean 6.55, SD 0.44), followed by those using the platform 4 to 6 times weekly (mean 6.05, SD 0.70), 2 to 3 times weekly (mean 5.82, SD 0.62), and once weekly (mean 5.20, SD 0.99). Bonferroni-adjusted post hoc comparisons indicated that participants who interacted with the platform once per week reported significantly lower TAS scores than those who used it 4‐6 times per week (*P*=.007) or daily (*P*<.001). Total minutes of use over the prior 4 weeks were positively associated with TAS overall scores (β=0.0015 per minute; SE=0.0006; *t*_63_=2.55, *P*=.01). These analyses should be interpreted as associative and exploratory rather than causal, because the temporal relationships between platform engagement and TAS scores cannot be determined. Additional descriptive analyses of TAS scores by caregivers’ characteristics are presented in Multimedia Appendix 6.

**Table 2.** Exploratory association between overall Technology Acceptance Survey score and platform interaction characteristics (N=65).

Variables	Value	TAS score (SD)	Test statistics	*P* value
Platform interaction frequency, n (%)			*F*_3,61_=7.88^a^	<.001
Daily	10 (15.4)	6.55 (0.44)		
4‐6 times per week	18 (27.7)	6.05 (0.70)		
2‐3 times per week	20 (30.8)	5.82 (0.62)		
Once per week	17 (26.1)	5.20 (0.99)		
Platform use (in minutes in the past 4 weeks), mean (SD)	187.0 (167.0)	5.83 (0.85)	β=0.0015, SE=0.0006; *t*_63_=2.55^b^	.01

a ANOVA.

b Linear regression.

### Qualitative Thematic Analysis Results

#### Overview

Aligned with our codebook, thematic analysis generated 3 major themes: challenges of caregiving, evaluation of the Olera platform, and feedback on AI-assisted support. The challenges of caregiving theme captured the emotional, interpersonal, and structural barriers that shaped participants’ experiences. The evaluation theme reflected caregivers’ perspectives on the usefulness, usability, and areas for improvement of the Olera platform. Finally, participants were asked about AI-assisted caregiving because of the recognized need for more customized support and our goal of developing AI-powered digital tools in the future.

#### Challenges of Caregiving

Caregivers described substantial barriers to care in their roles, characterized by emotional strain, social isolation, and the complexity of daily responsibilities. Feelings of loneliness and disconnection were salient, with one participant noting, “The isolation and loneliness [are the hardest], and my siblings who are not involved criticize everything I do” (PID0001). Similar sentiments were echoed by another caregiver who reflected, “Being understood by friends and family, in regard to the care of my father, has been very difficult” (PID0023). The demands of continuous caregiving were also underscored; one respondent explained, “Organizing everyday tasks is the most difficult part, trying to keep everything moving while balancing my own life feels impossible” (PID0007). In addition to the burden of responsibilities, caregivers frequently described interpersonal strain, including conflict with family members and resistance from care recipients. As one participant shared, “Trying to make decisions that my mother would have wanted was the most stressful part, especially when others disagreed” (PID0003). Respondents also highlighted the distress of witnessing progressive decline, stating, “Watching a loved one declining more each day was heartbreaking” (PID0009). Another respondent expressed a similar statement, saying that it was difficult “seeing change in them and seeing them disappear” (PID0152).

System-level barriers and financial challenges further complicated caregiving. As one participant explained,

Navigating the healthcare system can be a significant obstacle to getting a loved one with dementia the care they need. For example, it can be difficult to get approval for specialized dementia care or to understand insurance coverage; the stress of waiting on long waitlists or being turned down for services only increases.

[PID0007]

The same participant further noted financial difficulties that were a prevalent concern among caregivers, saying:

Financial difficulties are another prevalent problem; paying for memory care facilities or professional caregivers can be expensive, and families frequently have to make out-of-pocket payments due to government program coverage gaps. These obstacles make it necessary for many caregivers to provide all care for themselves, which causes emotional strain and burnout as they attempt to fulfill the requirements of both themselves and their loved ones.

[PID0007]

Another caregiver explained, “Financial issues combining households made it hard to afford professional care” (PID0023).

#### Evaluation of the Olera Platform

A majority of caregivers (96.9%, 63/65) evaluated the Olera platform positively, frequently emphasizing its helpfulness and relevance to their caregiving needs. Respondents described the caregiving resources and articles as practical and timely for their caregiving. For example, one caregiver stated, “The caregiving services and educational publications were good and very helpful” (PID0007). Another caregiver noted, “There was a very good variety of needed topics that were covered” (PID0027). Respondents highlighted the accessibility of the site and its ability to provide comprehensive and well-organized content, as illustrated by a participant who shared, “It was easy to move through the (web)site and I didn’t have trouble finding what I needed” (PID0025). Another wrote, “The website was good overall in all the categories, very informative” (PID0023). Another caregiver expressed appreciation for the breadth of resources: “I found them useful, and I wish I had it available to me years ago” (PID0075). Another caregiver expressed the helpfulness of the platform for caregivers with various levels of caregiving experience, saying: “The site is useful for new caregivers and seasoned caregivers” (PID0050).

Participants also identified several areas for improvement and further development. A total of 5 out of 65 participants noted encountering unexpected pages or slow page loading speed. As one respondent noted, “A couple times I clicked on a link and it did not take me where I expected” (PID0001) with another saying, “I found a couple of 404 s” (PID0109). Another respondent suggested, “Improving page load speed and link functionality would enhance overall usability” (PID0007). Caregivers also suggested additional features, including expanded resource listings with more affordable options and broader educational content, reflected in the statement, “I would like to see more resources for affordable care options, because many listed were far out of reach financially” (PID0023). These insights have already informed recent platform improvements, such as expanded filters for payment and coverage options, to better match users with affordable providers. Caregivers also mentioned the need for more specialized information for caregivers with different needs. As one participant noted, “More educational resources would help, especially customized information so people can get what they specifically need” (PID0016). Several expressed a need for more detailed content, saying*,* “Some topics could have gone into more detail” (PID0102), and another expressing the platform “need[s] much more depth, I pretty much knew what was there, but know there’s much more to learn” (PID0109).

#### Feedback on AI-Assisted Support

When asked about AI-assisted caregiving support services, participants (N=58) expressed either confidence or caution. Their open-ended responses indicated both openness to innovation and concerns regarding trust in data, accuracy, and limited understanding.

Among all participants (N=58) who answered the AI-assisted support questions, 65.5% (38/58) reported confidence in AI-assisted caregiving support and viewed AI as a promising tool to ease caregiving demands. For example, one participant explained, “I don’t lack confidence in this type of support. I’m in favor of anything that will make my current situation easier” (PID0105). Others conveyed optimism that AI could supplement their own efforts and provide tailored resources, with a caregiver noting, “Very confident in AI. I am always looking for more information on what I can do to improve my mom’s nutrition” (PID0079). These comments highlight receptivity to AI when it is perceived as directly alleviating caregiving burdens.

At the same time, 34.5% (20/58) of the respondents reported hesitancy and uncertainty about AI-assisted caregiving and expressed concerns regarding the accuracy of the data and privacy protection. Respondents reported a lack of knowledge of how to use AI and a preference for human interactions. One caregiver noted, “I don’t trust a computer to assist me. I want a human being that I can talk to and assist you” (PID0082). One respondent stated, “I would need more information and to get used to it first before I could trust it” (PID0023). Another respondent said, “AI assisted information is still so new that I don’t trust it yet” (PID0150). These responses suggest that while openness exists, many caregivers remain cautious and seek reassurance through exposure and education. Additionally, caregivers suggest that building confidence will depend on the confidentiality of information shared with AI and the accuracy of information provided by AI. As one caregiver explained, “Building confidence would require making sure the information is correct and that my data is kept private” (PID0007). Another emphasized the importance of transparency and verification of content, noting, “If I knew how the information was generated and that it came from reliable sources, I would trust it more” (PID0015). Another participant agreed with the sentiment, saying “If there were consistent footnotes to verify information provided, I would have more confidence in AI” (PID0131).

## Discussion

### Principal Findings

This study evaluated caregiver acceptance of the Phase II Olera web-based platform, an example of a beneficial personalized digital caregiving support tool. The platform was evaluated using adapted TAS measures and open-ended qualitative feedback. Overall, family caregivers reported an overall TAS score of 5.83 (SD 0.85) on a 7-point scale. Acceptance scores increased with frequency of use, suggesting an association between technology engagement and acceptance, with daily users reporting the highest TAS scores compared to once-weekly users (mean 6.55, SD 0.44, and mean 5.20, SD 0.99, respectively). The continuous iterative process of the Build-Measure-Learn development model is likely the contributing factor to the repeated high acceptance and usability scoring in our assessments [18].

Regarding opinions on the platform, participants described the platform as helpful, relevant, and easy to navigate. The caregivers also stated that the platform provided timely educational and service information for their caregiving needs. Caregiver feedback for future improvement of the platform included providing more affordable and geographically diverse resources, broader educational content, and more personalized information for specific caregiving needs. Thematic analysis of open-ended responses after use of the platform identified opportunities for further improvement of the platform, including suggestions such as more robust coverage of service providers caring for ADLs and information on how to afford or pay for care. Thematic analysis also reproduced common challenges of informal caregiving, including social isolation and daily responsibilities [8]. Participants often described difficulties managing financial barriers, such as high out-of-pocket costs for professional care, along with navigating fragmented health and insurance systems.

Caregivers expressed both enthusiasm and caution regarding AI-assisted support. Participants were open to AI tools helping them understand their caregiving needs, identify professional services, and use available financial aid to pay for care. Moreover, approximately two-thirds of participants expressed confidence in using AI-assisted tools for assisting caregiving. However, it should be noted that one-third of respondents reported hesitancy and expressed concerns about trust in AI, accuracy of the information provided by AI, privacy of information shared with AI, and lack of familiarity with AI.

### Comparison With Prior Work and Implications

Acceptance of caregiver-facing digital tools has been commonly assessed using validated instruments such as the TAS and the SUS [31,33,37]. In our study, the tested Phase II Olera platform achieved an overall TAS mean score of 5.83 (SD 0.85, 83% of the scale maximum) [56]. This level of acceptance is comparable to or higher than acceptance levels reported in other caregiver-facing tools. For example, the CARES app (Social Wellness, LLC and Emissary Health) reported SUS scores of 73‐77 (73%‐77% of scale maximum), interpreted as “good–excellent” usability [61], while the myHealthE caregiver portal (CAMHS Digital Lab), designed for caregivers in child and adolescent health, was scored lower, with a mean SUS of 62.4 (SD 15, 62% of scale maximum), categorized as “marginal–OK” usability [62]. Professional aides’ use of the Mobile Smart Care System yielded an average Unified Theory of Acceptance and Use of Technology score of 46.9/50 (SD 5.46, 94% of scale maximum), reflecting very high acceptance, though in a different user group [63]. These comparisons suggest that the Olera platform demonstrated strong caregiver acceptance, performing at least as well as, and in some cases better than, other recently evaluated caregiver-facing platforms. Qualitative feedback further supported the perceived usefulness and ease of use needed for more affordable, accessible, and context-specific resources, which have broader implications for digital caregiving intervention design and considerations.

Our finding is that platform use frequency is positively associated with the TAS score. This finding suggests that it is essential to design features to encourage regular interaction, such as onboarding sessions, ongoing training, and reminders to users to encourage digital health adoption. Additionally, while not statistically significant, we observed a trend between the rate of technology adoption and technology acceptance levels wherein participants who adopted technologies earlier than most of their peers or were among the first to try new tools reported higher acceptance levels of the Olera platform. This suggests that early technology adopters may be somewhat more receptive to new digital tools, though further research is needed to confirm this pattern.

Although our sample size was insufficient for reliable comparisons across racial and ethnic groups, prior studies that show variability in technology comfort and adoption across demographic groups, such as Bratches et al [64], who reported that non-Hispanic Black or African American caregivers were less likely than non-Hispanic White caregivers to feel comfortable using smartphones for caregiving tasks; and Larnyo et al [65], who highlighted the influence of socioeconomic status and technology access on disparities among African American users. Broader evidence on the digital divide similarly shows that minority caregivers and people with disadvantaged socioeconomic status may encounter barriers to both access and confidence in digital health use [66]. Given the limited sample size, further research should examine caregiving technology acceptance in a more representative population. Moreover, participants in this study reported a good proficiency with technology overall, indicating a possible higher confidence level and decreased barrier to technology use at baseline.

Regarding caregiving challenges, our findings were consistent with the literature documenting the multidimensional burdens of dementia caregiving, particularly within the United States [2,8,67,68]. Caregivers in our study emphasized emotional strain, social isolation, and the complexity of navigating fragmented health systems, often compounded by financial hardship. These findings aligned with our prior study that dementia caregivers experience psychological distress, financial strain, and have difficulties in securing services [8]. Other US-based studies also suggested that health system fragmentation exacerbates out-of-pocket spending and delays in accessing memory care, thereby contributing to caregiver burnout and reduction of quality of life for both caregivers and their people living with dementia [69]. These systemic barriers suggest that conventional support mechanisms are insufficient to address the complex needs of dementia caregivers, and innovative, accessible solutions are urgently needed.

While our study highlights the attitude of informal caregivers of people living with dementia toward AI use in technological assistive tools, it is important to note that these are more descriptive, exploratory findings. With AI increasingly positioned as a potential tool to personalize caregiving [24,25], our findings regarding AI acceptance highlight both promise and caution. Most caregivers in our study expressed openness to AI, particularly when it was framed as a way to reduce information burden, streamline access to fragmented services, and provide tailored caregiving strategies. This finding is consistent with emerging evidence showing that conversational agents and digital assistants can promote caregiver well-being by facilitating access to relevant educational resources and mitigating social isolation [24]. At the same time, caregivers in our study expressed concerns about accuracy, transparency, and confidentiality, echoing broader evidence of public skepticism toward AI in health care settings [26-28]. Prior work has emphasized that the perceived reliability of AI outputs and assurance of data privacy are critical determinants of trust and adoption [69].

Furthermore, these findings align with the AI Trust Framework and Maturity Model, which positions the trustworthiness of an AI system along 7 pillars: explainability, data privacy, robustness and safety, transparency, data use and design, societal well-being, and accountability [70]. Our cohort’s concerns of accuracy, transparency, and confidentiality, as well as greater trust in beneficial AI, reflect the AI Trust Framework and Maturity Model’s key pillars. Caregivers’ concerns with transparency distinctly highlight one pillar. Additionally, concerns of accuracy can be addressed by explainability and accountability, while confidentiality is addressed by data privacy, robustness and safety, and data use and design. Finally, societal well-being is demonstrated by caregivers’ greater openness to using AI that serves a purpose, which in their case is reducing burden and isolation. Therefore, while AI has the potential to mitigate systemic barriers and alleviate caregiving burdens, its successful implementation will require sustained efforts to build trust through reliable content, transparent design, cultural sensitivity, and caregiver-centered education and training. These findings will help inform the development of our future iterations of the Olera platform.

### Strengths and Limitations

This study has several notable strengths. First, the use of a mixed methods design enabled a more comprehensive evaluation of the Olera platform by integrating quantitative survey data with qualitative feedback, allowing for data triangulation and deeper insights into caregivers’ experiences. This approach not only captured usability and acceptance but also identified concrete areas for ongoing refinement of the platform. Second, the study sample reflected a diverse caregiver profile in terms of socioeconomic background, race and ethnicity, and geographic distribution, aligning well with national caregiving demographics [1], thereby supporting the relevance of our findings.

At the same time, several limitations should be considered in interpreting the results. While there were some participants (n=5) who were previously exposed to the Olera platform, their one-time usage of the previous version was not considered a confounding variable, as there were subsequent updates to the platform and they were not exposed to the website for a prolonged period of time. The recruitment sample size was smaller than originally planned despite extensive efforts to broaden outreach [17]. Despite the decreased sample size, the final sample included meaningful representation across racial and ethnic groups, including an “Other” category that included Asian, Native American or Alaska Native, and multiracial caregivers. The retention rate was 57% (65/114), which reflected the common attrition challenges in remote digital health studies [71]. Although no statistically significant differences were observed between completers and noncompleters, individuals with heavier caregiving burdens (eg, more weekly caregiving hours, role strain or conflict) were more likely to discontinue participation, which may have limited generalizability to the most burdened caregivers. This may also present a gap for future platform development in the usability of this digital platform for higher-burdened caregivers. As such, dedicated measures should be taken to incentivize or support the continued participation of caregivers with heavy time constraints in future studies. Additionally, our sample predominantly consisted of women from the United States (61/65, 95.3%), who reported high levels of technological literacy. This may be attributable to the reported majority of dementia caregivers being women who contribute more time to caregiving and, therefore, may experience greater levels of burden [1]. As such, the possibility that female caregivers have a greater need or interest in caregiving research may have influenced our study sample. Future efforts should be taken to recruit a larger male population to increase the generalizability of results. Finally, while our platform was developed so caregivers with elementary-level comprehension could use it, we were unable to recruit those with lower literacy to test its usability. This likely presents a digital literacy bias since our cohort demonstrated relatively high levels of health and digital literacy, which may not reflect the broader population of caregivers, particularly those with lower literacy or more limited access to technology. Thus, while the findings provide valuable evidence of the platform’s acceptance and utility, they should be interpreted with these limitations in mind.

### Future Platform Development and Work

In the next phase of platform development, we plan to incorporate a step-by-step user interface and user experience orientation to key user features that mimics the walkthrough given on our orientation calls, which were found to be helpful for users. Based on the qualitative and quantitative data obtained in this study, we plan to prioritize further refinement of (1) the navigation menu bar to help families more quickly compare providers and establish care relationships by developing more nuanced filters based on provider payment and coverage options; (2) the current quiz that is automatically initiated at account creation to become more personalized and include questions about benefits, insurance, and out-of-pocket budget; and (3) filters for various services, including tagging long-term services and support with benefits, coverage, and accepted payment methods. These features will aid informal caregivers in more efficiently finding information and services that are also personalized to their needs and financial barriers.

Regarding the findings surrounding the use of AI in navigating the eldercare ecosystem, we plan to develop an eldercare-specific large language model (LLM) that is trained to personalize service recommendations, estimate out-of-pocket costs, and screen families for eligibility for financial benefits that can help pay for professional caregiving services. To address caregiver hesitancy in adopting AI-assisted digital tools, our LLM training protocol will incorporate Reinforcement Learning from Human Feedback, which is a model training method that aligns AI with human preferences by using human judgments to guide its output [72]. The initial prototype will be evaluated and its parameters adjusted based on the feedback from experts in the caregiving field, including licensed clinicians and social workers, and further refined through usability assessments with informal caregivers according to our established Build-Measure-Learn product development framework.

### Conclusions

Our findings support prior research that caregivers face systemic challenges that conventional support mechanisms are insufficient in addressing, indicating the urgent need for easily accessible and innovative solutions. A web-based platform that is characterized by minimal design, informativeness, interactivity, ease of use, and usability, and provides tailored suggestions on caregiving information and services that address caregivers’ needs is more likely to be well accepted by informal dementia caregivers. Informal caregiver feedback throughout the development of the platform is essential. Future research and iterations of the Olera platform are essential to enhance the tool to better alleviate caregiving burdens across more diverse caregivers at different stages of dementia care.

## Supplementary material

10.2196/92967 Multimedia Appendix 1 Demonstration of the Olera website during the study.

10.2196/92967 Multimedia Appendix 2 Emails and text messages used for communication and sharing of website material during the study.

10.2196/92967 Multimedia Appendix 3 Emails and text messages used for recruitment communication.

10.2196/92967 Multimedia Appendix 4 Technology Assessment Survey adapted from the Technology Assessment Model.

10.2196/92967 Multimedia Appendix 5 Codebook for thematic analysis of qualitative data in Technology Acceptance Survey.

10.2196/92967 Multimedia Appendix 6 Extended table of perceived acceptance of technology by all participant characteristics collected (N=65).

10.2196/92967 Multimedia Appendix 7 Comparison of characteristics between participants who completed the study and those lost to follow-up.

10.2196/92967 Multimedia Appendix 8 Mean and SD of Technology Acceptance Survey items (N=65).

10.2196/92967 Multimedia Appendix 9 Distribution of Technology Acceptance Survey responses (N=65).

## Abbreviations

AD: Alzheimer disease
ADL: activity of daily living
AI: artificial intelligence
CONSORT: Consolidated Standards of Reporting Trials
LLM: large language model
SUS: System Usability Scale
TAM: Technology Acceptance Model
TAS: Technology acceptance survey
