# Supplementary material for: Exploring Informal Caregivers’ Perception of the Olera Digital Caregiving Assistance Platform for Dementia Care: Mixed Methods Evaluation Study
Source: JMIR Form Res. 2026 Jul 3;10:e92967. doi: 10.2196/92967 (PMC13331331; doi:10.2196/92967)

**Olera.care website platform design during the study**

Website link: <https://olera.care/>

Home page:


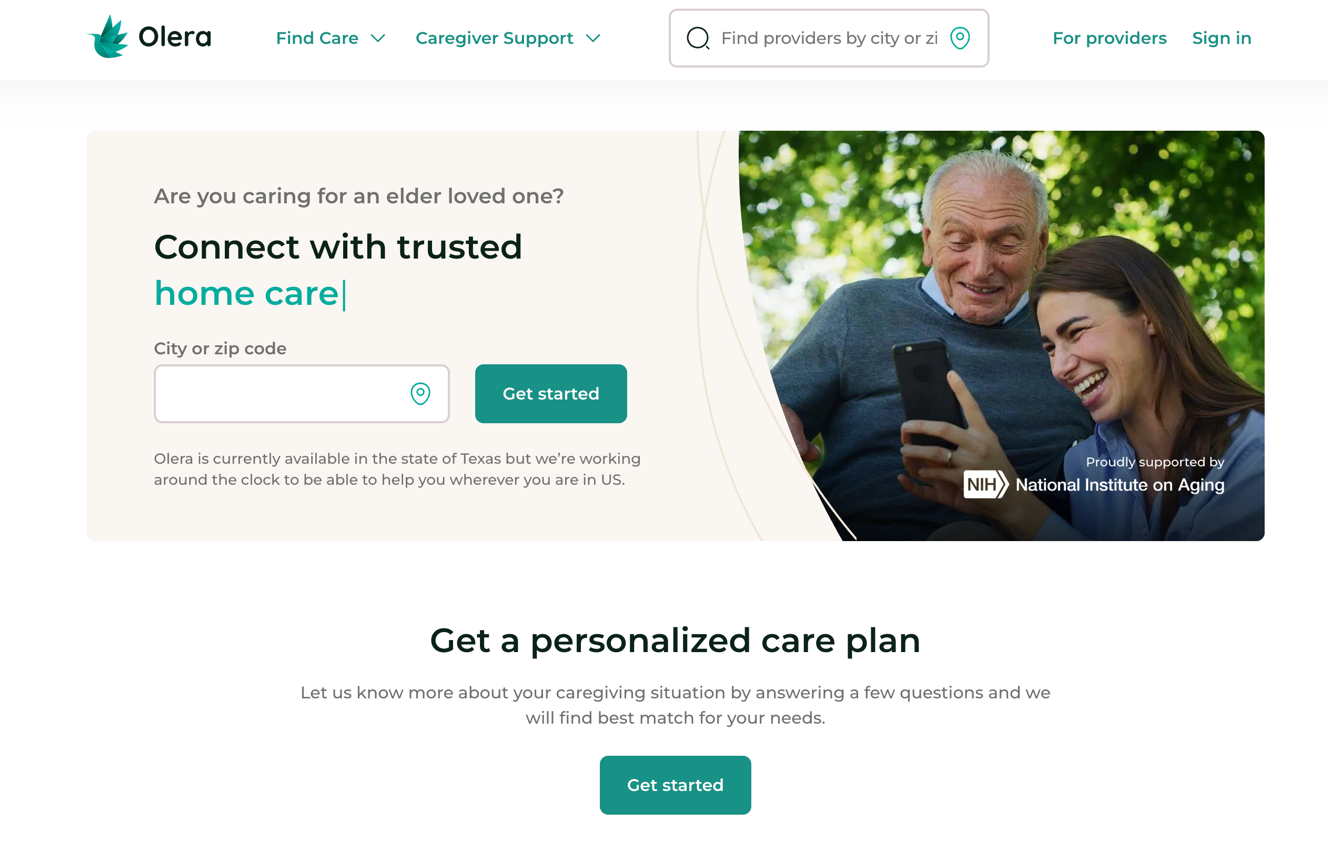


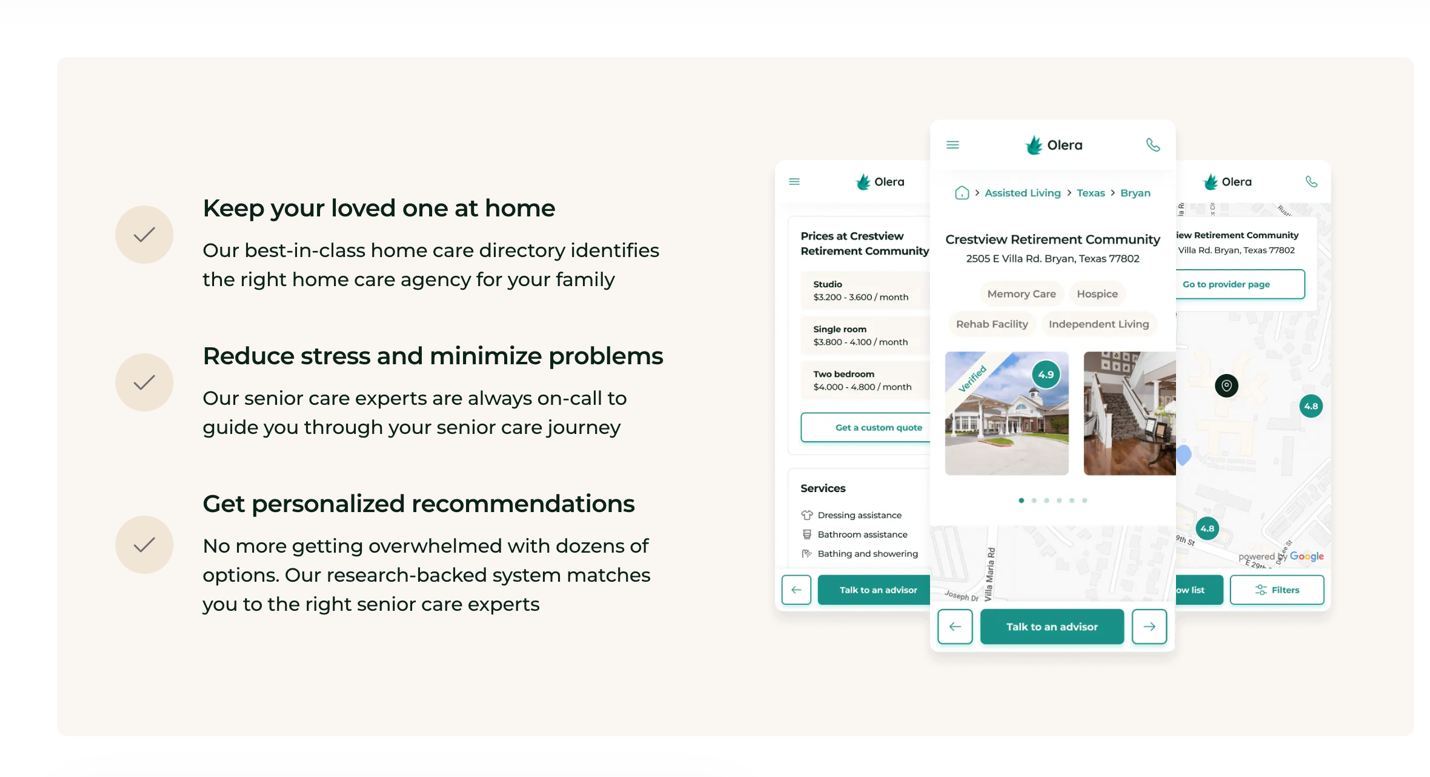


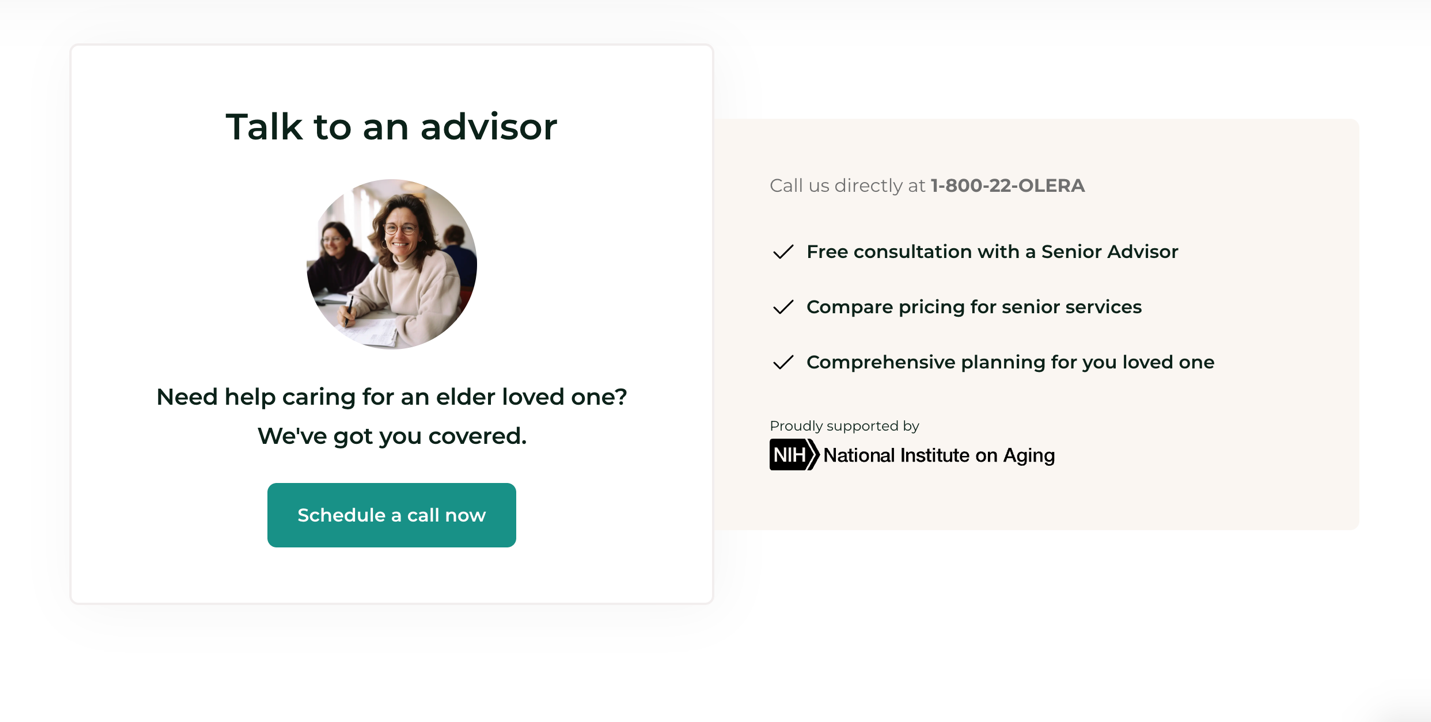

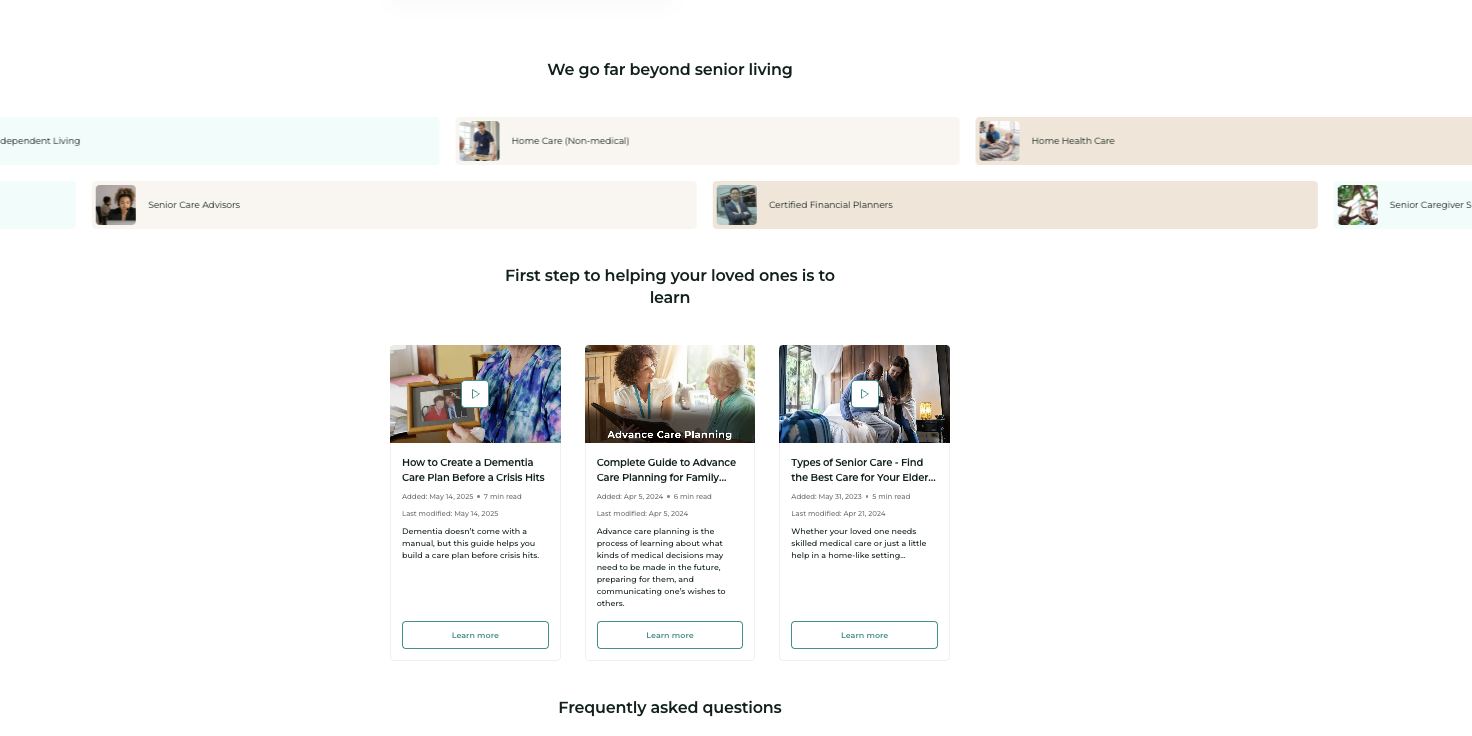


Account creation page and initial personalization quiz:


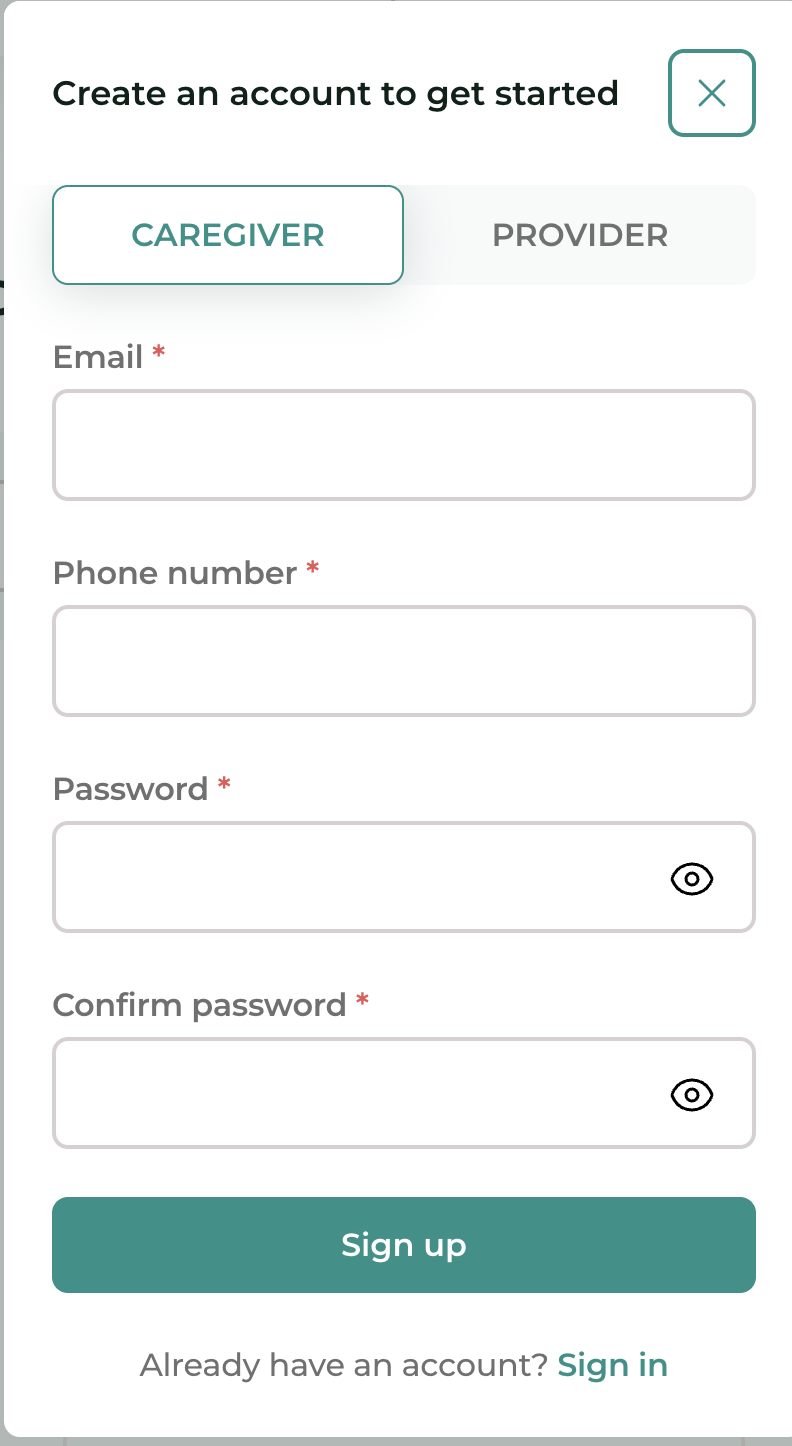


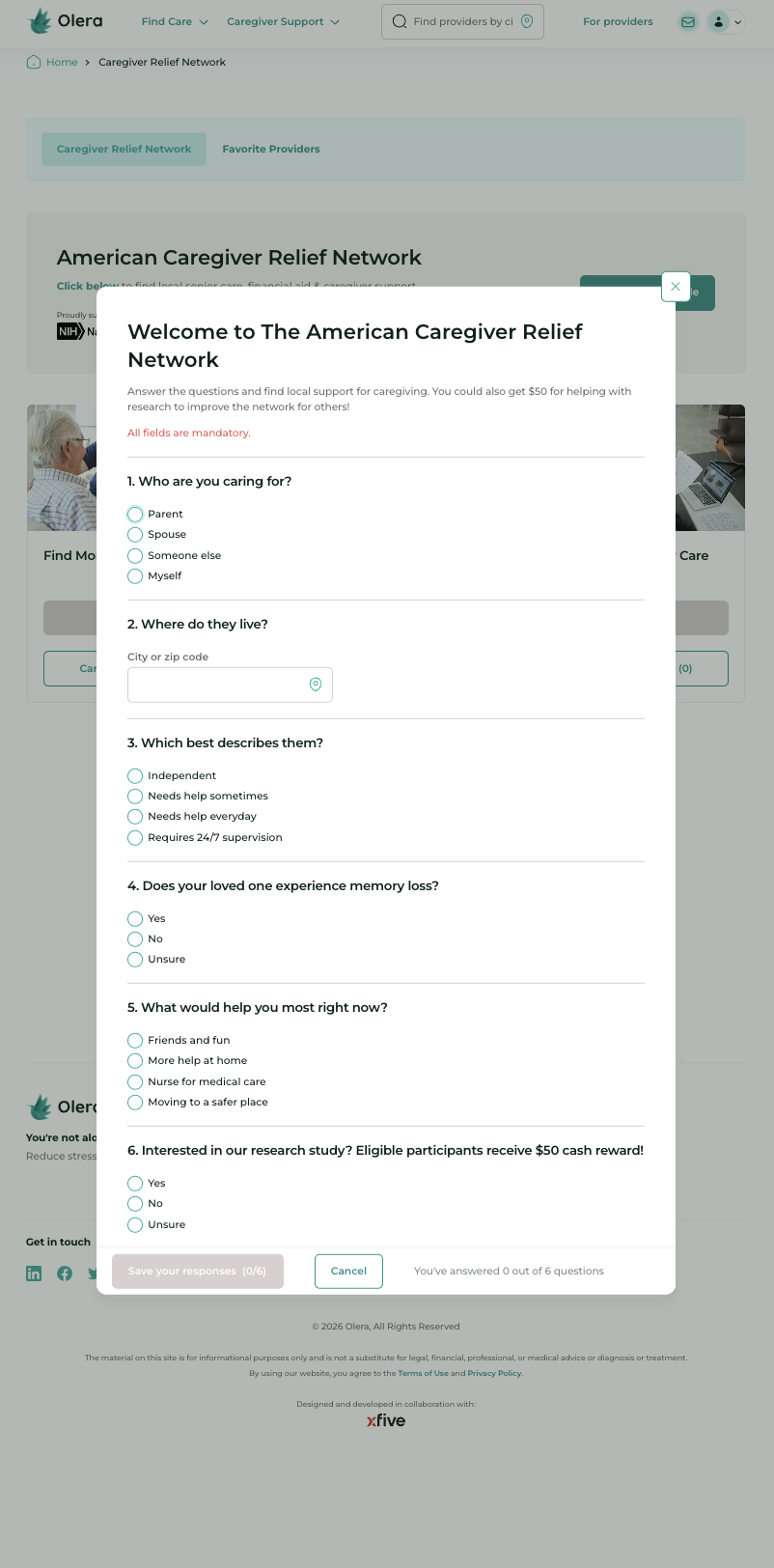


Example of user’s Caregiver Support Network:


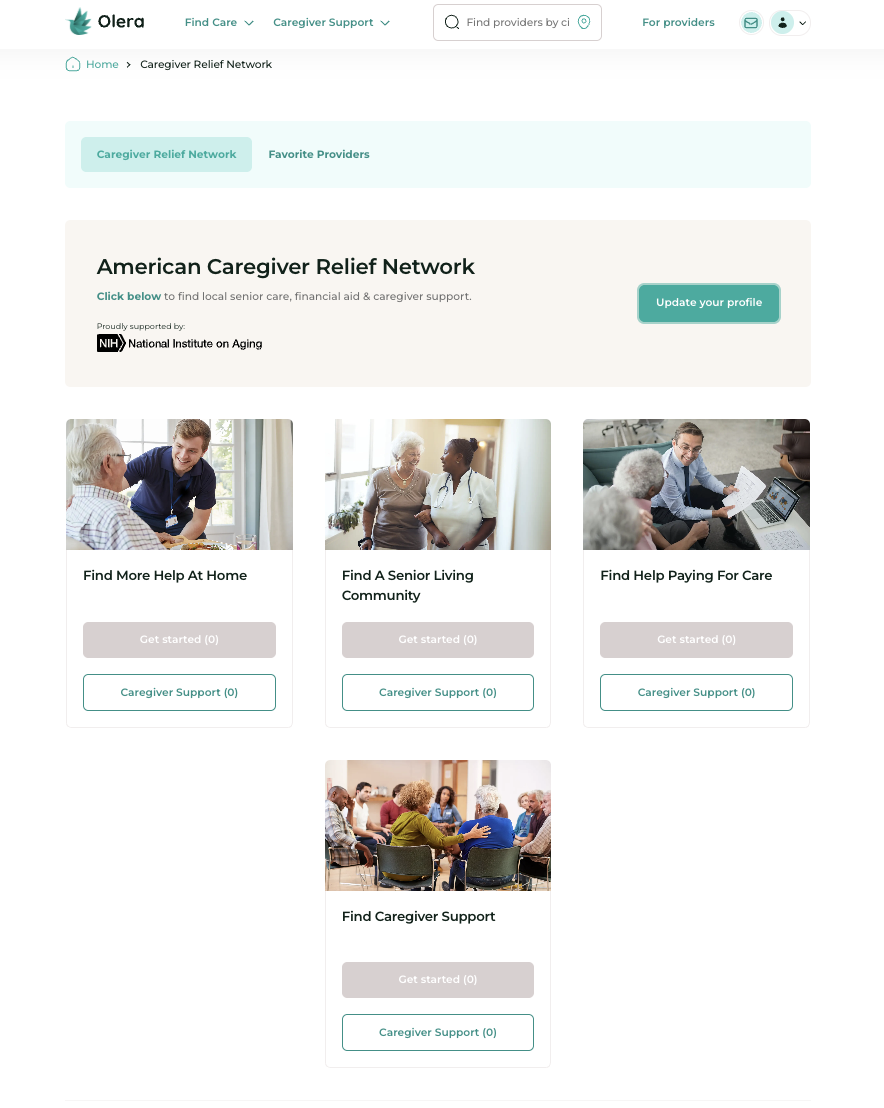


Example of resources finder for a caregiver based on Zip code and current caregiving needs:


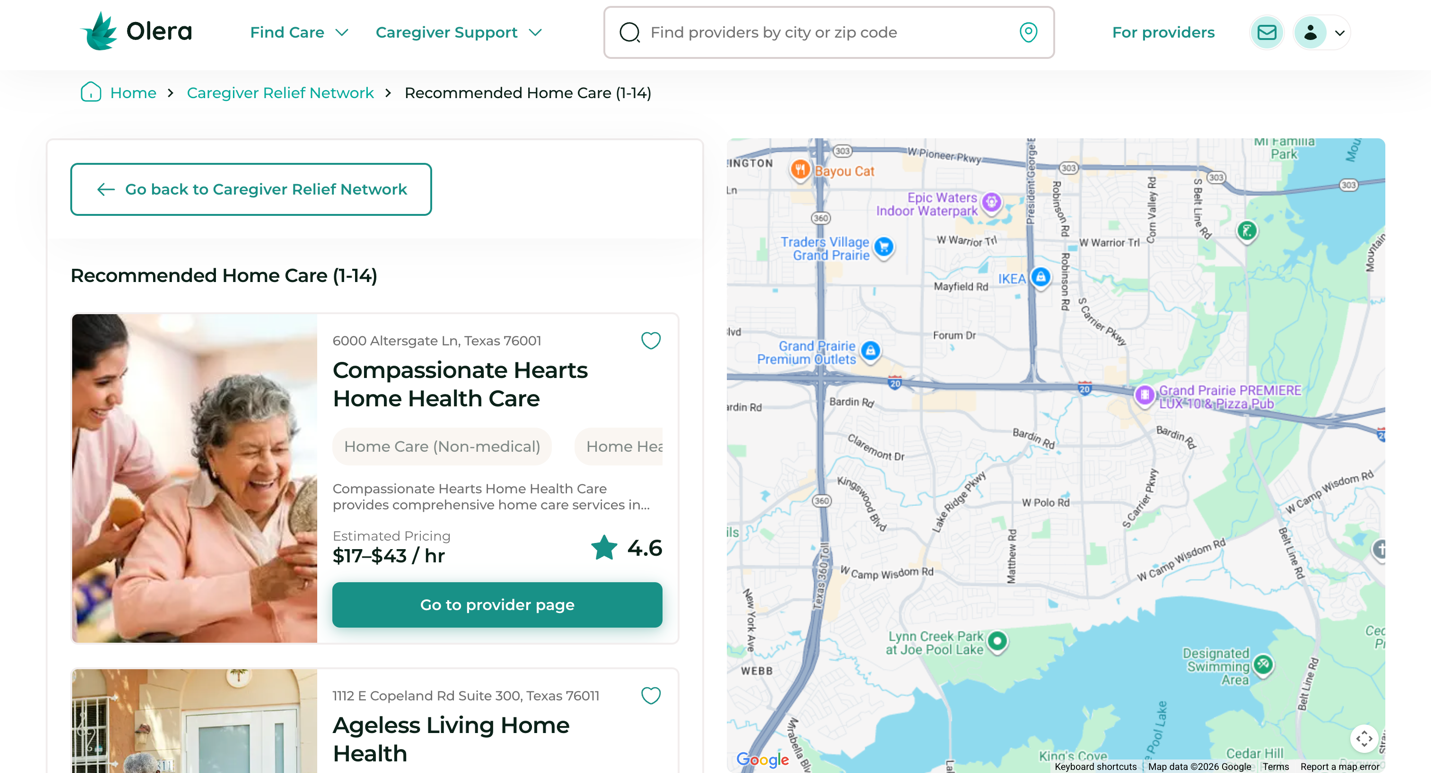


Example of articles available on the website:


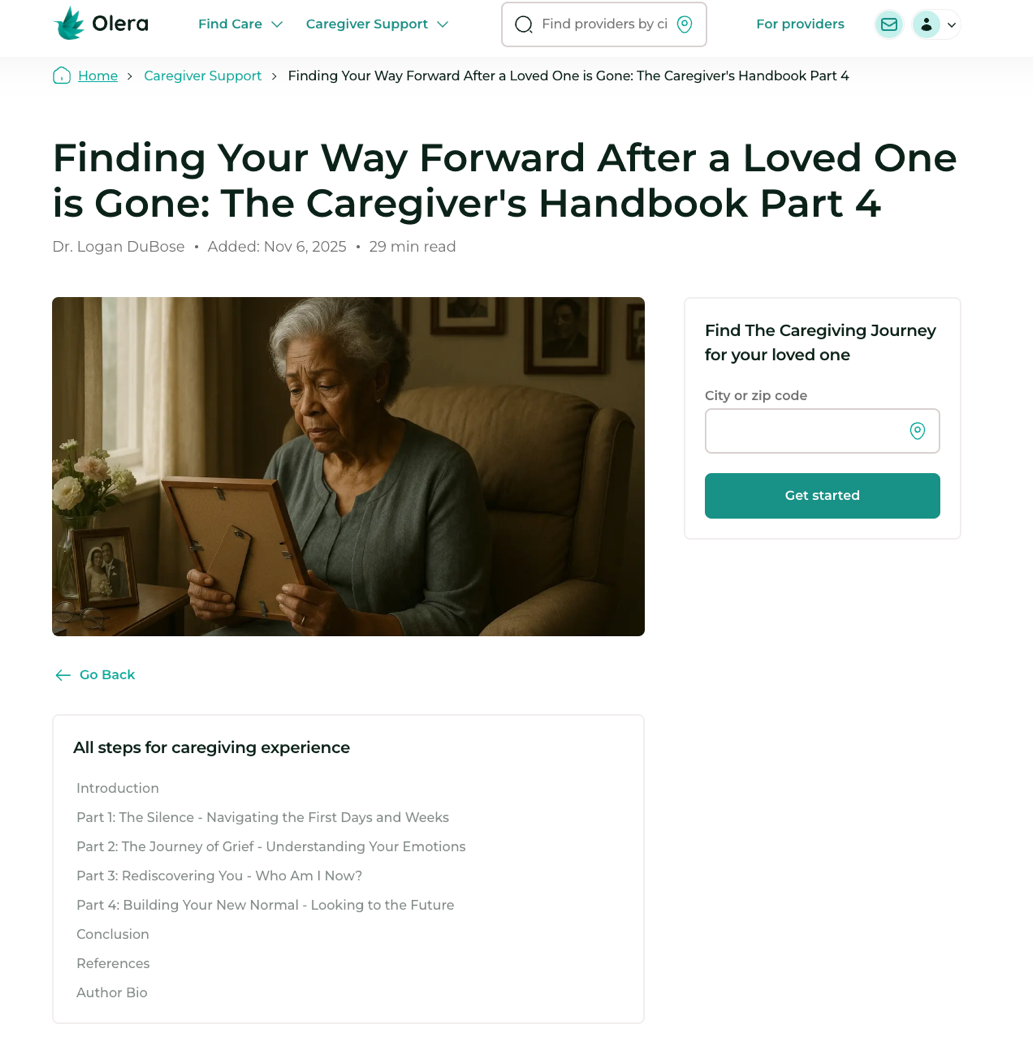


Example of Caregiver Community Forum:


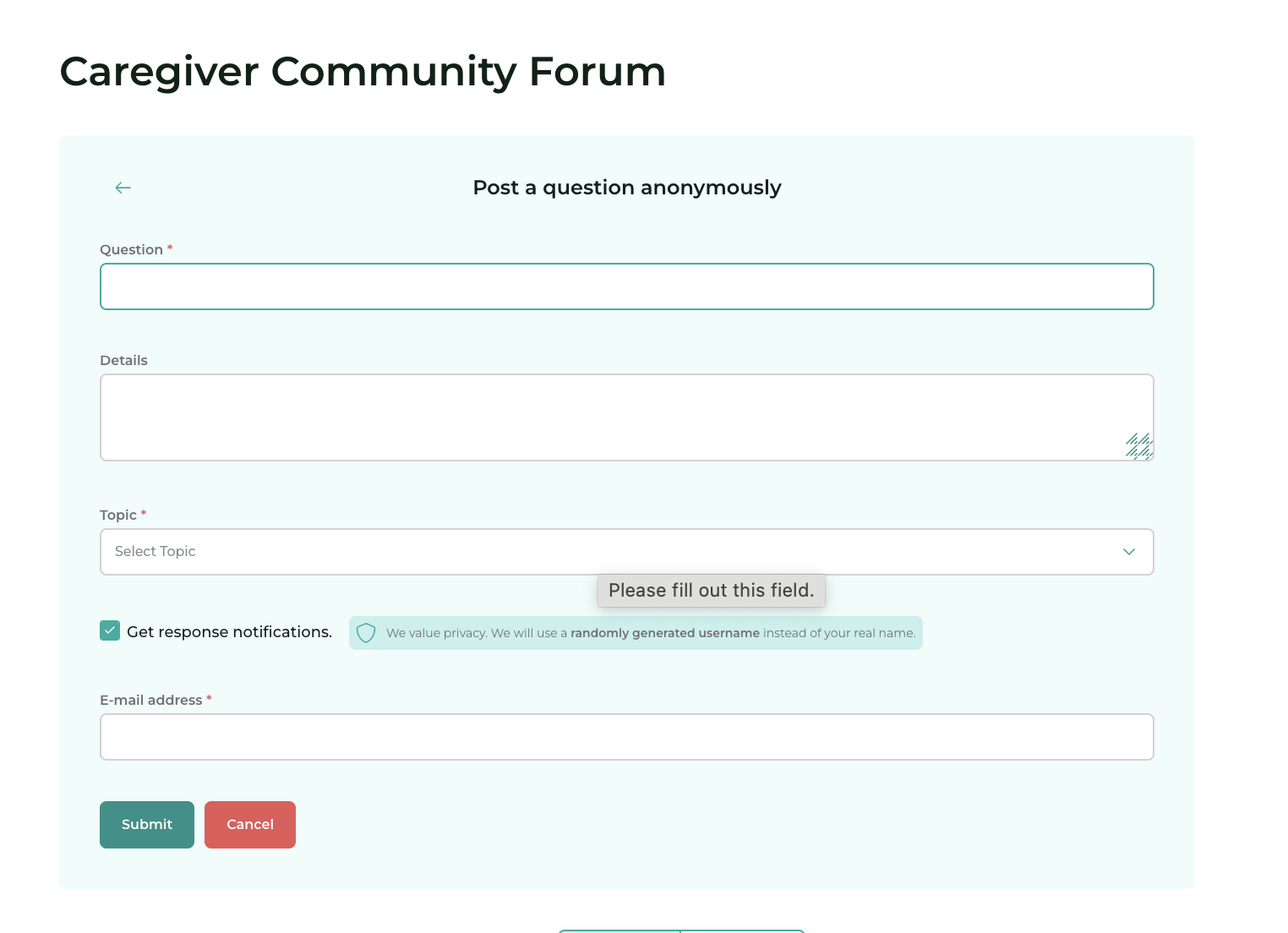


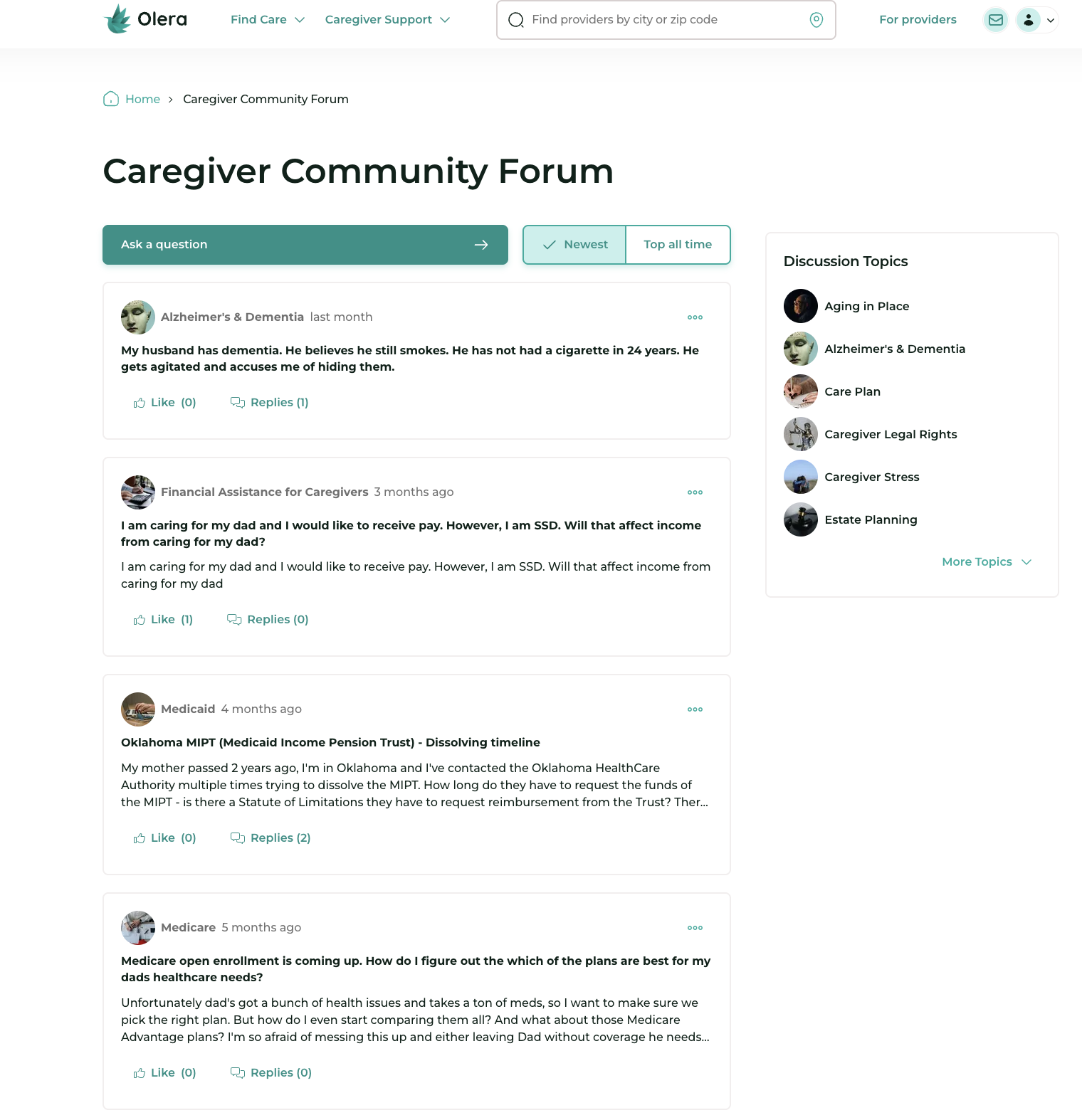

Supplement: Multimedia Appendix 1 [file formative-v10-e92967-s001.docx]
