# Supplementary material for: Exploring Informal Caregivers’ Perception of the Olera Digital Caregiving Assistance Platform for Dementia Care: Mixed Methods Evaluation Study
Source: JMIR Form Res. 2026 Jul 3;10:e92967. doi: 10.2196/92967 (PMC13331331; doi:10.2196/92967)
Supplement: Multimedia Appendix 2 [file formative-v10-e92967-s002.docx]

**Supplemental File 1: Texts and Emails Used for Study Procedures**

1. **Example of the Flow of Automated Communication Sequences with Participants via Zapier**


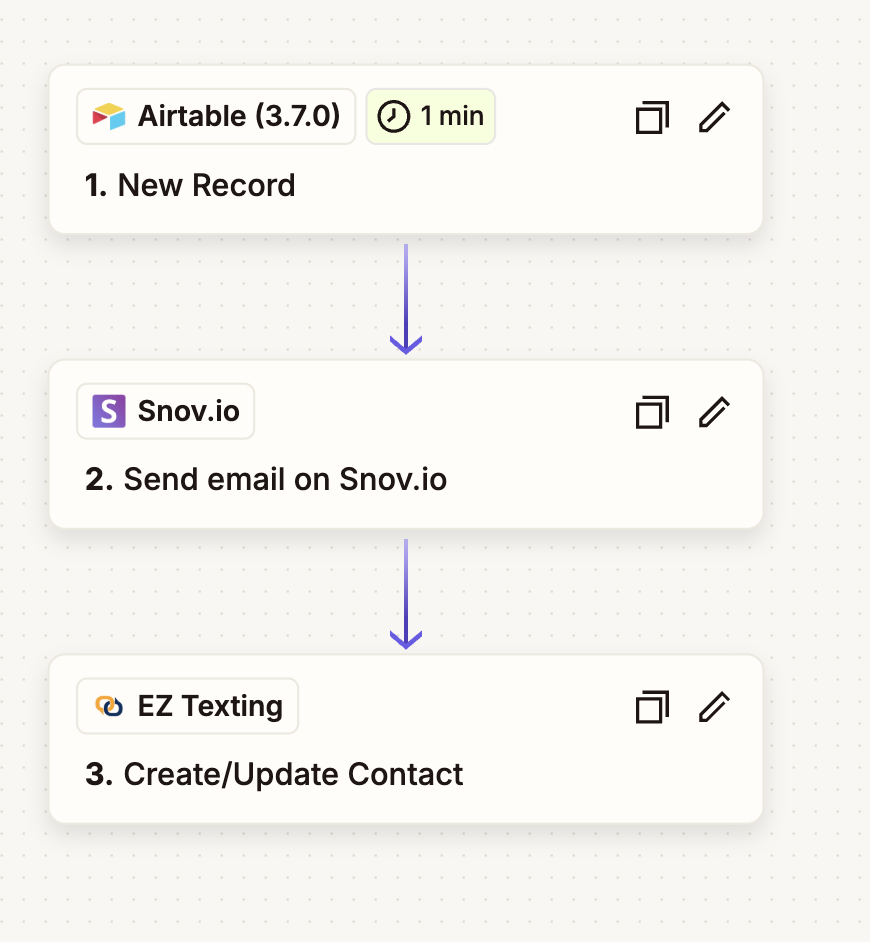


1. **Overview of the Email Sequence to Prompt Interaction with the Olera Platform Flow Snov.io**


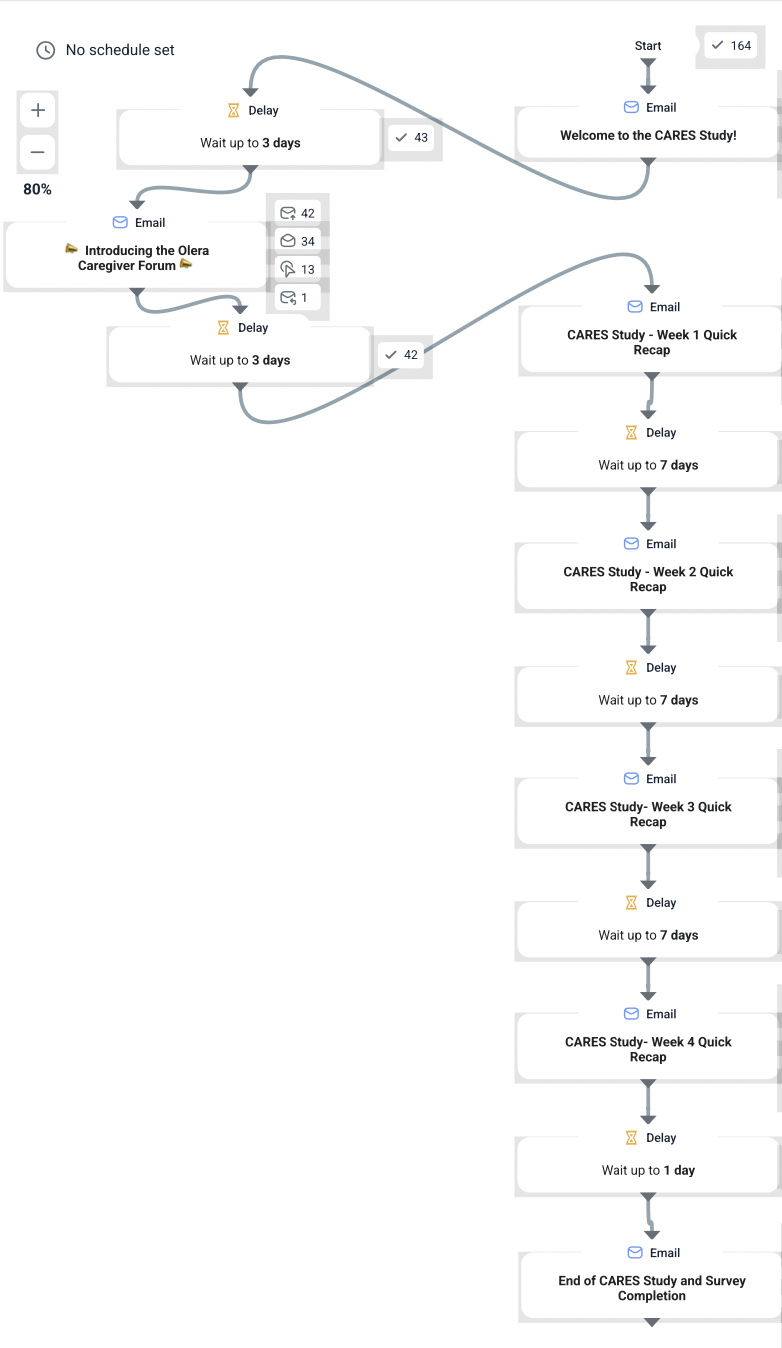


1. **Content of Weekly Emails Sent to Participants to Prompt Interaction with the Olera Platform via Snov.io**
2. Welcome email:

Subject line: **Welcome to the CARES Study!**

Welcome!

We welcome you,{full_name}, to the Caregiver Assistance, Resources, and Education For Seniors (CARES) study! Your participation is needed for us to improve our caregiver website.

There are only 3 steps to complete the study:

- **Step 1.** Log [In here](https://olera.care/) each day to use our website over the next four weeks.
- **Step 2.** Interact with the Olera website and take notes on your opinions about the website.
- **Step 3.** Complete the Technology Assessment Survey at the end of the four weeks.

Here's what you need to know:

- **Purpose of the Study:** The CARES study is about providing caregivers with support, information, and resources. We aim to empower you with knowledge and tools to make the caregiving experience more manageable and fulfilling.
- **Informative Website, Articles, and Videos:** Over the coming weeks, we will invite you to use our website to learn about various caregiving topics, from legal aspects to practical tips for providing care. We will also send you articles, videos, and surveys to get your opinions on the quality and usefulness of the content.
- **Low Time Commitment:** You can spend as little as 5 minutes a day reading an article or looking at the information on our website.
- **Your Critiques:** Your feedback is invaluable to us as we strive to improve our website. Please **write down any feedback you have on our website's design, quality, usefulness, and function** over the next four weeks.
- **Final Exit Survey & $50 CASH REWARD:** Complete all the surveys we send, and we'll give you $50 CASH REWARD once your participation and responses are verified!
- **Optional Exit Zoom Session:** Schedule an optional 30 minute Zoom session with us where you can share your thoughts on the Olera platform.

We look forward to having you in the study! Please complete the steps above and contact us with any questions.

**Your participation in the CARES study is entirely voluntary, and you can opt out at any time by texting or emailing “STOP.” However, we encourage you to stay engaged and take advantage of the wealth of knowledge and support that will be available to you.*Best,

1. Second email three days after welcome email:

Subject line: **📣 Introducing the Olera Caregiver Forum 📣**

Good morning {full_name| caregiver},

**Introducing our brand new Caregiving Community.**

This forum is designed for you to post or answer questions *anonymously* to get help or help other caregivers in need. Take a look around and explore the topics, read other caregiver’s experiences and questions, or share your own.

**Please post on our Caregiver Community or reply to another caregiver’s question through the link:**

[**Olera Caregiver Community**](https://olera.care/caregiver-forum?utm_source=snov&utm_medium=email&utm_campaign=GenCRM_Cohort1&utm_content=main_link)

Any feedback is appreciated, and if you can make a post, it would be a huge help as we get the community going! Thank you again!

Best,

1. Summary email at end of week 1:

Subject line: **CARES Study - Week 1 Quick Recap**

Dear {first_name | caregiver},

We hope this email finds you well and that you've had a productive week! As part of the Caregiver Assistance, Resources, and Education For Seniors (CARES) study, we have been sending you helpful information about caregiving.

Here is a quick recap of the caregiving information we have sent you so far:

**Planning Ahead for Senior Care** 📖📆

Proper planning is essential to ensure the well-being of your loved ones and to alleviate the burden of caregiving. If you haven't already, take a moment to read the article and watch the video: [Read Article](https://olera.care/caregiver-support/paying-for-senior-living-exploring-finance-options-and-assistance-programs)**Want to ask other caregivers for advice? Post in our forum 💬 🤔**

Want to share your experiences or get advice from other family caregivers? Make a post on our forum about your caregiver questions or experiences to help yourself and other caregivers who may be in a similar situation: [Olera Caregiver Community](https://olera.care/caregiver-forum?utm_source=snov&utm_medium=email&utm_campaign=GenCRM_Cohort1&utm_content=main_link)

**Connect with Caregiver Support Groups** 🤝

As we near the end of the week, we want to remind you that you are not alone on this caregiving journey. You can search for Support Groups near you using this link: [Find Support Group
_____   _____   _____   _____   _____   _____   _____   _____   _____   _____   _____   ________________](https://olera.care/caregiver-support/caregiver-support-groups-finding-local-and-online-groups)

We encourage you to set aside some time to explore these resources and gain a deeper understanding of the topics discussed this week. Our goal is to empower you with knowledge and resources that can make your caregiving experience more manageable!

Remember, you can always visit our website for even more information and resources related to caregiving. If you have any questions or need further assistance, please get in touch with us. [Login In Here.](https://olera.care/caregiver-relief-network)

Thank you for being a part of the CARES study, and we look forward to continuing this journey with you. Have a wonderful day and happy reading!

Best,

1. Summary email at end of week 2:

Subject line: **CARES Study - Week 2 Quick Recap**

Dear {first_name | caregiver},

We hope this email finds you well and that you've had a productive week! As part of the Caregiver Assistance, Resources, and Education For Seniors (CARES) study, we have been sending you helpful information about caregiving.

Here is a quick recap of the caregiving information we have sent you so far:

**Understanding Different Senior Living Options** 🏡

Proper planning to ensure your loved one is in the best living situation is essential. If you haven't already, take a moment to read the article: [Read Article](https://rpocarservice.com/track/click/v2-524079539)**Finding Help with Senior Care Insurance 🤝🏻**

Finding an insurance agent may help. Read this short article about what to look for when finding an insurance agent: [Read Article](https://rpocarservice.com/track/click/v2-524079545)

**Do you ever feel lonely as a caregiver? Connect with other caregivers in our forum 💬**

As we near the end of the week, we want to remind you that you are not alone on this caregiving journey.

Reach out to other caregivers via our forum about your personal struggles as a caregiver. Your experience can help other caregivers: [Olera Caregiver Community](https://rpocarservice.com/track/click/v2-524079549)

[_____](https://rpocarservice.com/track/click/v2-524079552) [_____](https://rpocarservice.com/track/click/v2-524079553) [_____](https://rpocarservice.com/track/click/v2-524079554) [_____](https://rpocarservice.com/track/click/v2-524079555) [_____](https://rpocarservice.com/track/click/v2-524079556) [_____](https://rpocarservice.com/track/click/v2-524079557) [_____](https://rpocarservice.com/track/click/v2-524079558) [_____](https://rpocarservice.com/track/click/v2-524079559) [_____](https://rpocarservice.com/track/click/v2-524079560) [_____](https://rpocarservice.com/track/click/v2-524079561) [___ _ _ _ _ _ _ ____](https://rpocarservice.com/track/click/v2-524079562) [_____](https://rpocarservice.com/track/click/v2-524079563)

We encourage you to set aside some time to explore these resources and gain a deeper understanding of the topics discussed this week. Our goal is to empower you with knowledge and resources that can make your caregiving experience more manageable!

Remember, you can always visit our website for even more information and resources related to caregiving. If you have any questions or need further assistance, please get in touch with us. [Login In Here.](https://olera.care/caregiver-relief-network)

Thank you for being a part of the CARES study, and we look forward to continuing this journey with you. Have a wonderful day and happy reading!

Best,

1. Summary email at end of week 3:

Subject line: **CARES Study- Week 3 Quick Recap**

Dear {first_name | caregiver},

We hope this email finds you well and that you've had a productive week! As part of the Caregiver Assistance, Resources, and Education For Seniors (CARES) study, we have been sending you helpful information about caregiving.

Here is a quick recap of the caregiving information we have sent you in week 3:

**Adult Daycare Services** 🏡

Finding temporary care for your loved one could help improve your loved one's quality of life and provide you with a much-needed break or time to run your errands. Here's an article to learn more about these services: [Read Article](https://olera.care/caregiver-support/adult-daycare-centers-a-guide-to-choosing-the-right-service-for-your-loved-one)

**Respond to another caregiver's forum post** 🤔

Have you responded to a caregiver's post in the forum? Your experience is invaluable and can help another caregiver in need. Respond to another caregiver's post today: [Olera Caregiver Community](https://olera.care/caregiver-forum?utm_source=snov&utm_medium=email&utm_campaign=GenCRM_Cohort1&utm_content=main_link)

**Creating a Personal Care Agreement** 📃 ✍🏿

Creating a Personal Care Agreement with your loved one could qualify you to be paid as a family caregiver through Medicaid. Here is a guide that explains what a Personal Care Agreement is and whether you should create one with your loved one: [Read Article](https://olera.care/caregiver-support/compensating-family-caregivers-understanding-medicaid-friendly-personal-care-agreements)

[_____   _____   _____   _____   _____   _____   _____   _____   _____   _____   _____   ______ _ _ _ _ _ _ _](https://olera.care/caregiver-support/caregiver-support-groups-finding-local-and-online-groups)

We encourage you to set aside some time to explore these resources and gain a deeper understanding of the topics discussed this week. Our goal is to empower you with knowledge and resources that can make your caregiving experience more manageable!

Remember, you can always visit our website for even more information and resources related to caregiving. If you have any questions or need further assistance, please get in touch with us. [Login In Here.](https://olera.care/caregiver-relief-network)

Thank you for being a part of the CARES study, and we look forward to continuing this journey with you. Have a wonderful day and happy reading!

Best,

1. Summary email at end of week 4:

Subject line: **CARES Study- Week 4 Quick Recap**

Dear {first_name | caregiver},

We hope this email finds you well and that you've had a productive week! As part of the Caregiver Assistance, Resources, and Education For Seniors (CARES) study, we have been sending you helpful information about caregiving.

Here is a quick recap of the caregiving information we have sent you in week 4:

**Finding Local Service Assistance Programs** 🏠

Finding local service assistance programs for your caregiving needs can be overwhelming. If you haven't already, take a look at some Olera-recommended resources ✅: [Read Article](https://olera.care/caregiver-support/family-caregiver-support-finding-local-resources-and-assistance-programs-near-you)

**Are you trying to find daily activities activities for your loved one? 🏏**

Are you having trouble finding daily activities for your loved one? If you need some ideas, or have some to share with other caregivers, head to our forum today to share your thoughts and questions. You could get responses from other caregivers and respond to other's with you *invaluable* experience. [Olera Caregiver Community](https://olera.care/caregiver-forum?utm_source=snov&utm_medium=email&utm_campaign=GenCRM_Cohort1&utm_content=main_link)

**Coordinating Medical Care with Senior Care Advisors** 🏥

Senior Care Advisors can help coordinate your loved one's medical care. Learn more about care coordinators here: [Read Article](https://olera.care/caregiver-support/finding-a-senior-care-advisor-a-guide)

[_____   _____   _____   _____   _____   _____   _____   _____   _____   _____   _____   ______ _ _ _ _ _ _ _](https://olera.care/caregiver-support/caregiver-support-groups-finding-local-and-online-groups)

We encourage you to set aside some time to explore these resources and gain a deeper understanding of the topics discussed this week. Our goal is to empower you with knowledge and resources that can make your caregiving experience more manageable!

Remember, you can always visit our website for even more information and resources related to caregiving. If you have any questions or need further assistance, please get in touch with us. [Login In Here.](https://olera.care/caregiver-relief-network)

Thank you for being a part of the CARES study, and we look forward to continuing this journey with you. Have a wonderful day and happy reading!

Best,

1. Concluding email request TAS completion:

Subject line: **End of CARES Study and Survey Completion**

Dear {full_name | caregiver},

**Thank you for interacting with the Olera Digital Platform for the past 4 weeks.** We truly appreciate your participation in the Caregiver Assistance, Resources, and Education for Seniors (CARES) study!

As a final step in the study , **please provide feedback on your experience with the Olera platform over the last 4 weeks through the Technology Assessment Survey through this link:**

[**Technology Assessment Survey Link**](https://tamu.qualtrics.com/jfe/form/SV_4IumMYIgfPj7Yge)

**If you would like to meet with our research staff to discuss your feedback via Zoom, feel free to schedule through this link:**[**Zoom Scheduling Link**](https://calendly.com/caregivers979/cares-study-zoom-exit-interview)**.**

If you have any questions before our final meeting, please feel to respond to this email with any questions or concerns.

Thank you for joining us in the CARES study, and we cannot wait to meet with you soon to hear your feedback on the Olera Digital Platform.

Best,

1. **Overview and Content of the Text Sequence to Prompt Interacting with the Olera Platform Flow via EZ Texting**


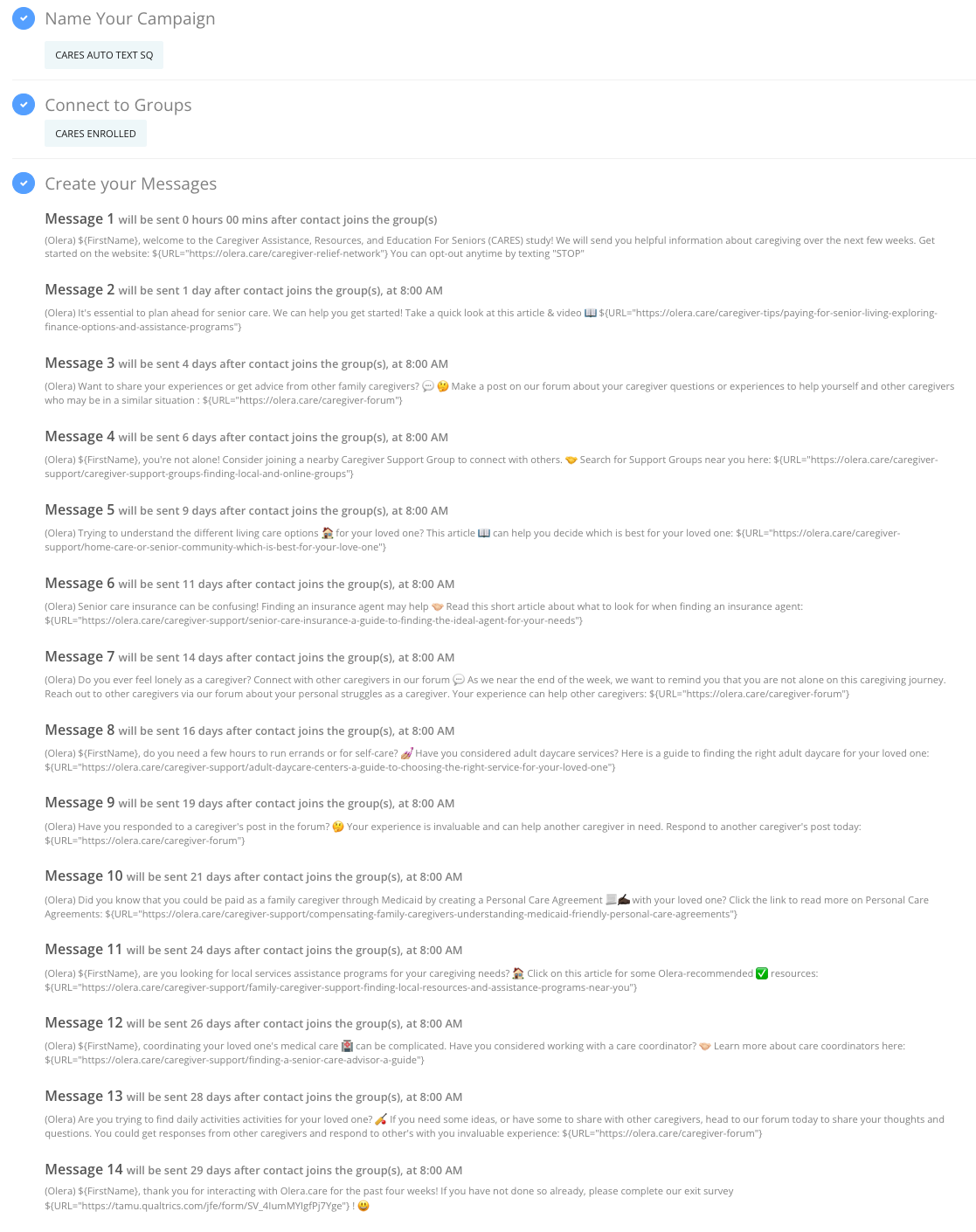


1. **Overview of the Flow of Automated Follow-Up for TAS Responses via Email**


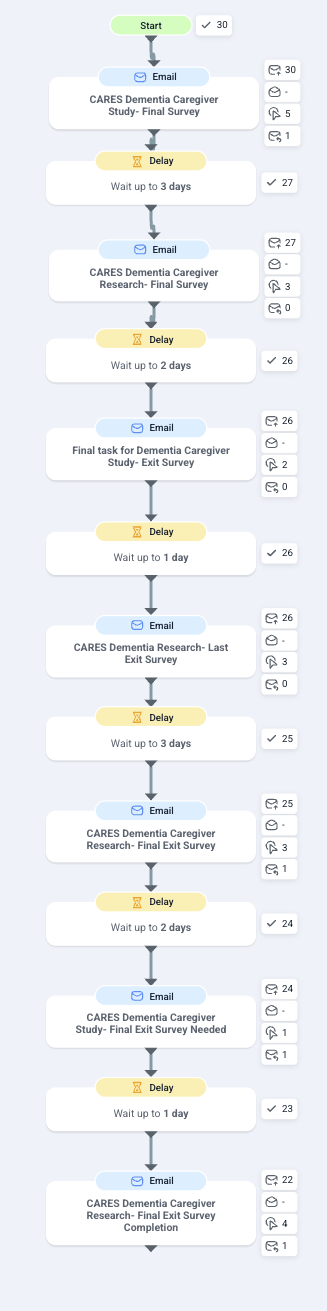


1. **Content of Emails Sent to Participants for TAS Responses via Snov.io**

The subject lines of each email were changed as seen above (5). The content of the email remained the same in all seven of the emails as seen below:

Hello {full_name | caregiver},

Thank you so much for your participation in our CARES study! Your participation is **invaluable** to us! I just checked our forms, and there are a few steps left to be completed in order to complete our study:

1. Complete the [**CARES Study- Technology Acceptance Survey**](https://tamu.qualtrics.com/jfe/form/SV_4IumMYIgfPj7Yge)

Our [Olera](https://olera.care/) website and services are available to you at any time! **To stay updated on Olera, be sure to like our Facebook page:** [Olera Facebook Page](https://www.facebook.com/oleracare/)

**If you would like to meet with our research staff to discuss your feedback via Zoom, feel free to schedule through this link:**[**Zoom Scheduling Link**](https://calendly.com/caregivers979/cares-study-zoom-exit-interview)**.**

If you have any questions before our final meeting, please feel to respond to this email with any questions or concerns.

Thank you again for your participation and feedback in our CARES study!

Best,

1. **Overview and Content of the Text Sequence Sent to Participants for TAS Responses via EZ Texting**

**
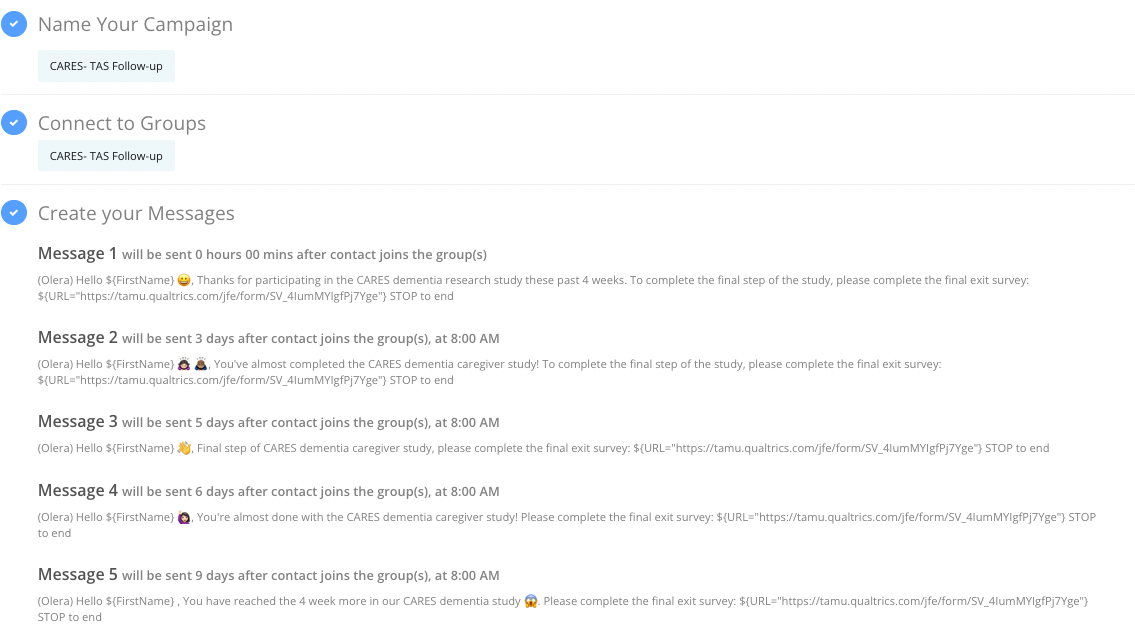
**

1. **Overview of the Flow of Automated Follow-Up for Details for Compensation via Email**

**
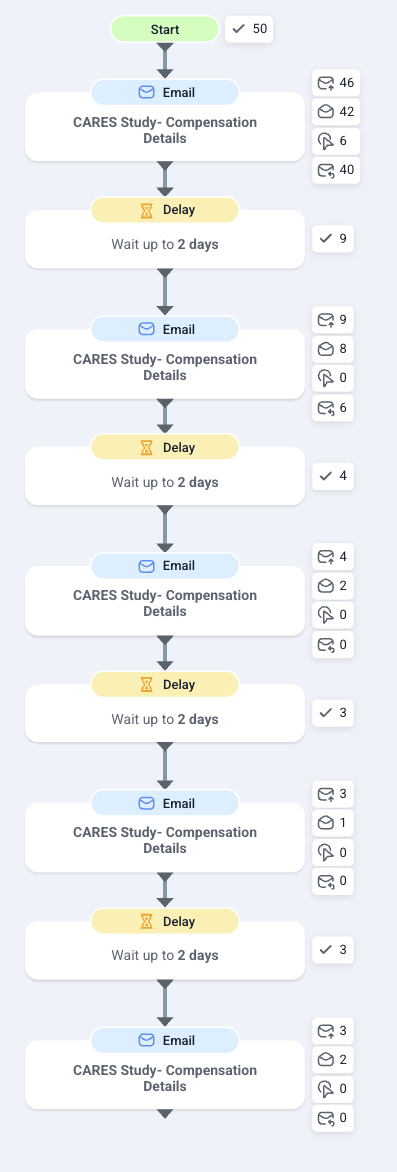
**

1. **Content of Emails Sent to Participants for for Details for Compensation via Snov.io**

The subject lines of each email were changed as seen above (8). The content of the email remained the same in all seven of the emails as seen below:

Good morning!

Thank you again for your participation in our CARES study these past 4 weeks. I just checked our forms, and it looks like the last survey was completed. Your feedback is invaluable as we continue to improve our website and study. **You will be receiving your stipend via check in the mail in the coming week.**

**To complete this study, could you please provide us with the following information:**

- **1. CARES stipend**: To receive your stipend, could you please send us your full name and good address to send the check to in the following format:
- Your full name
- Address: Street number and name, Apt/ suite # (if applicable)
- City, State Zip Code

Our [Olera](https://olera.care/) website and services are available to you at any time! **To stay updated on Olera, be sure to like our Facebook page:** [Olera Facebook Page](https://www.facebook.com/oleracare/)

Thank you again for your participation and feedback in our CARES study!

Best,

1. **Overview and Content of the Text Sequence Sent to Participants for Details for Compensation via EZ Texting**

**
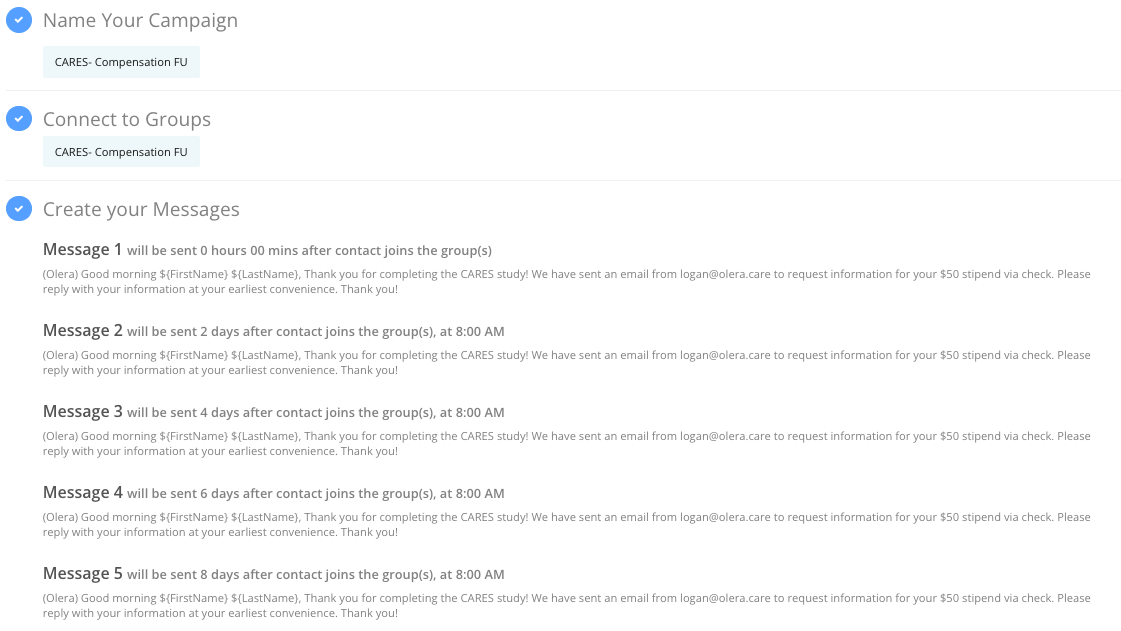
**
