# Supplementary material for: Exploring Informal Caregivers’ Perception of the Olera Digital Caregiving Assistance Platform for Dementia Care: Mixed Methods Evaluation Study
Source: JMIR Form Res. 2026 Jul 3;10:e92967. doi: 10.2196/92967 (PMC13331331; doi:10.2196/92967)
Supplement: Multimedia Appendix 3 [file formative-v10-e92967-s003.docx]

**Supplemental File 2: Emails and Text Messages Used for Recruitment**

1. **Example of the Flow of Automated Communication Sequences with Participants via Zapier**


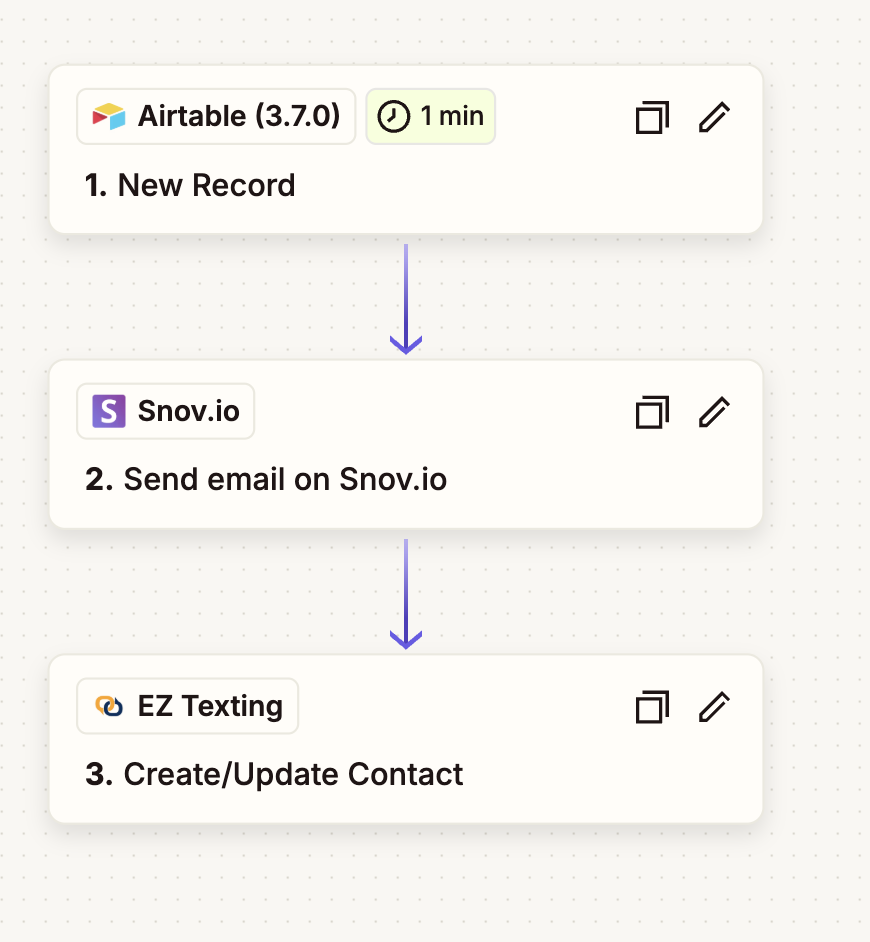


1. **Overview of the Recruitment Email Sequence to Facebook Leads via Snov.io**

**
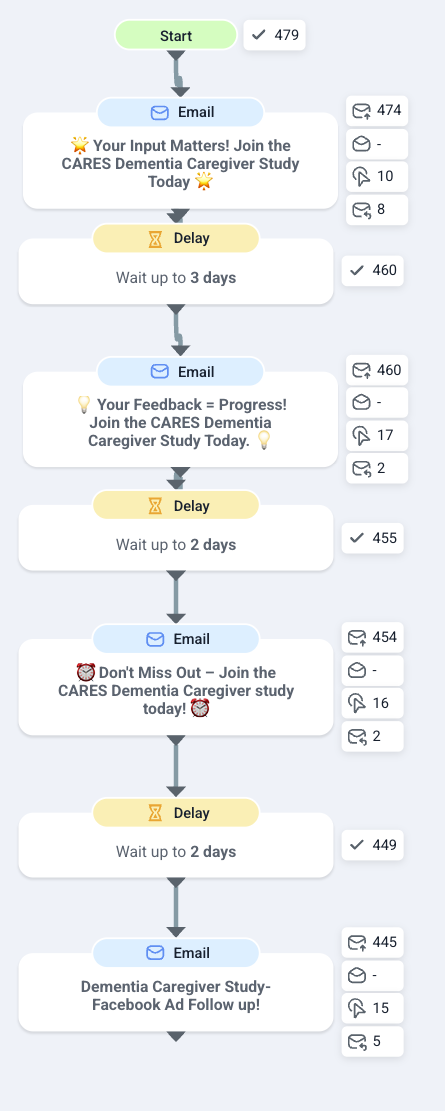
**

1. **Content of Recruitment Emails Sent Facebook Leads via Snov.io**

The subject lines of each email were changed as seen above (2). The content of the email remained the same in all seven of the emails as seen below:

Good morning,

If you have already completed the Qualtrics Intake Survey for the CARES Study, please disregard this message.

Thank you for your interest in the CARES Study for dementia caregivers via our Facebook ad. Your participation is invaluable for refining the Olera Digital Platform, offering tailored recommendations on dementia caregiving services and education.

To check your eligibility, please take a moment to complete our survey. Your responses will be confidential and used solely for research.

[**CARES Study Intake Survey**](https://tamu.qualtrics.com/jfe/form/SV_6QcBin4FTk8QKEK)

Your input will help us tailor support programs for caregivers like you, making a difference in their lives.

Please expect a call from us in the next day or two after filling out the survey.

Thank you for your commitment to advancing dementia care.

Warm regards,

**Note: Text message automations were not set up for initial Facebook leads due to the high volume and costs.**

1. **Overview of the Recruitment Email Sequence to Leads After Initial Recruitment Calls via Snov.io**

**
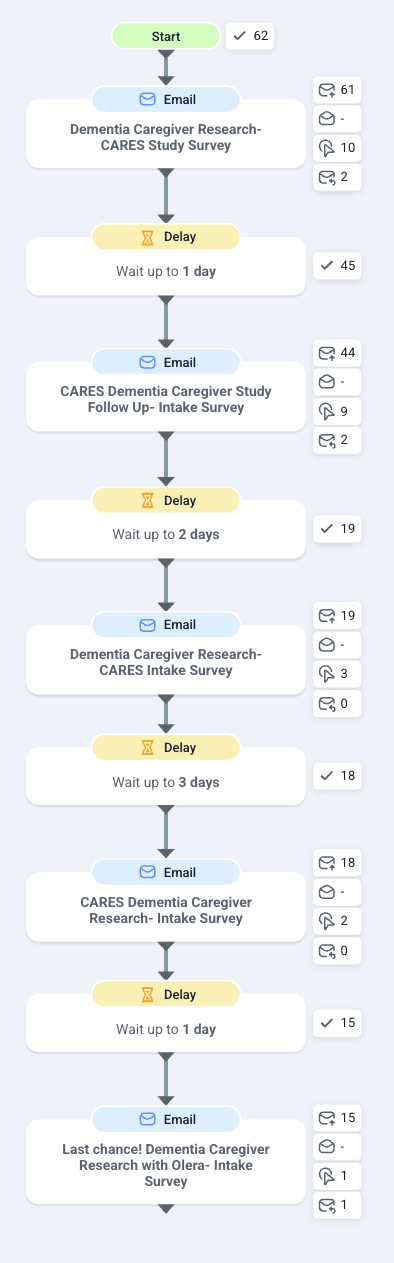
**

1. **Content of Recruitment Email Sequence to Leads After Initial Recruitment Calls via Snov.io**

The subject lines of each email were changed as seen above (4). The content of the email remained the same in all seven of the emails as seen below:

Hello {full_name |caregiver},

Thank you for talking to our research assistant about the CARES dementia caregiver study.

**To check your eligibility, please take a moment to complete our survey below.** Your responses will be confidential and used solely for research.

[**CARES Study Intake Survey**](https://tamu.qualtrics.com/jfe/form/SV_6QcBin4FTk8QKEK)

I have also attached a copy of the Informed Consent that has all the information you need to know about the study for your convenience.

Please let me know if you have any questions. Thank you for your time!

Best,

**Note: Text message automations were not set up for recruitment call leads due to the high volume and costs.**

1. **Overview of the Email Sequence to Leads to Create an Olera Account via Snov.io**

**
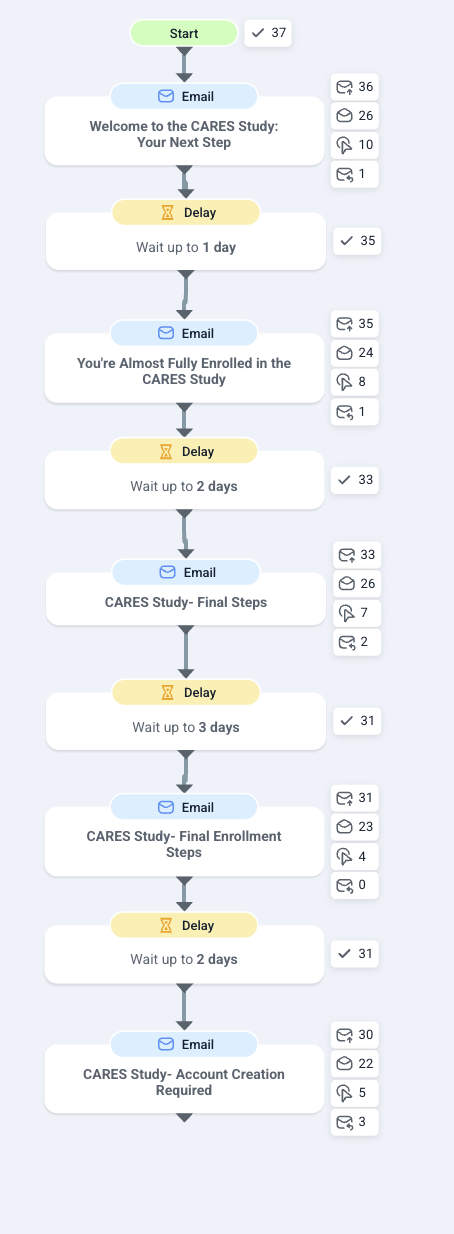
**

1. **Content of Recruitment Email Sequence to Leads to Create an Olera Account via Snov.io**

The subject lines of each email were changed as seen above (6). The content of the email remained the same in all seven of the emails as seen below:

Good morning {full_name | caregiver}, 

I hope this email finds you well.

Firstly, thank you for enrollowing *Caregiver Assistance, Resources, and Education For Seniors (CARES)*Study. Your participation is **invaluable** and will contribute significantly to our website development.

There is one last step to complete before you are fully enrolled: creating an account on our Olera Digital Platform so you can use the website and engage in our community.

**Please follow these steps to create your account:**

- Visit [[https://olera.care/](https://urldefense.com/v3/__https:/olera.care/__;!!KwNVnqRv!HwZn9jMzq0O1zg15YB-bSj9E5_uTzwG9Iy8yhWaPeqYLDgfiiXV_2oZBiDuYRGmHLQpXNkUEp8qaXlWJ4RuGCoSk0I6wesY$)]
- Click on the "***Sign In***" button on the top right of the page, then click***"Sign Up"***.
- Fill in the required information, ensuring accuracy.
- Once your account is created, you'll have access to exclusive content tailored to your needs and preferences.

Once you have completed this step, you will be fully enrolled in our CARES study! Over the next few weeks, you will be receiving emails and text recommendations for articles we believe may be helpful.

If you encounter any difficulties during the account creation process or have any questions, please don't hesitate to reach out to our support team at logan@oleracare.com. We're here to assist you every step of the way.

Thank you once again for choosing to be part of the CARES Study. Together, we are making a difference.

Warm regards,

1. **Overview and Content of the Text Sequence Sent to Leads to Create an Olera Account via EZ Texting**

**
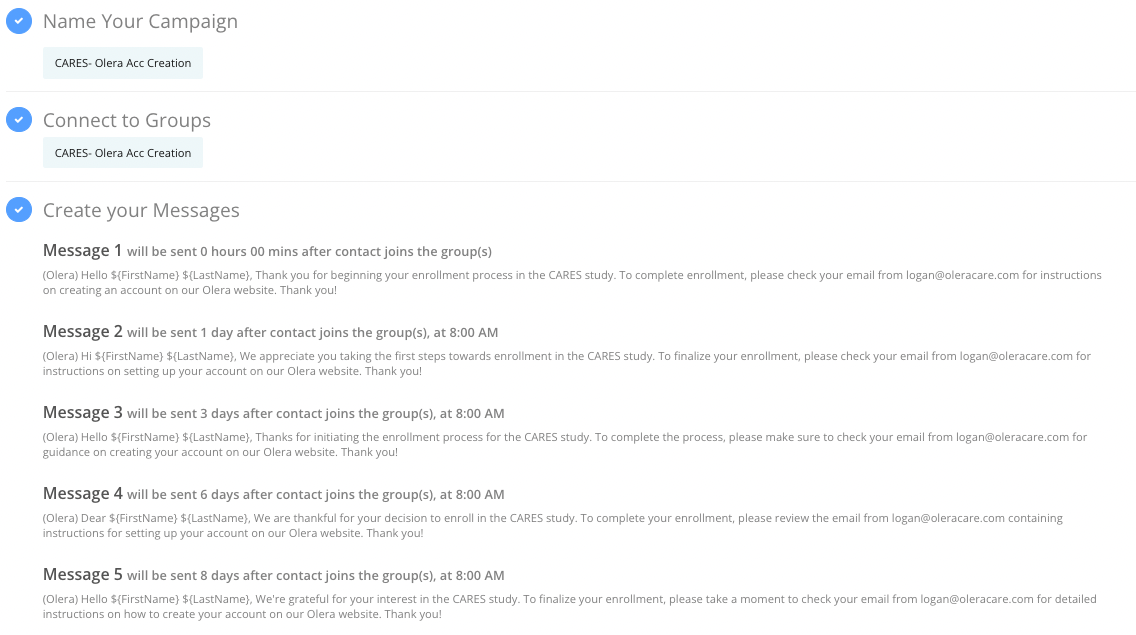
**
