# Supplementary material for: Exploring Informal Caregivers’ Perception of the Olera Digital Caregiving Assistance Platform for Dementia Care: Mixed Methods Evaluation Study
Source: JMIR Form Res. 2026 Jul 3;10:e92967. doi: 10.2196/92967 (PMC13331331; doi:10.2196/92967)
Supplement: Multimedia Appendix 4 [file formative-v10-e92967-s004.docx]

**Technology Acceptance Survey**

Thank you for your participation in the **CARES Study** the past four weeks.

Please answer the questions in this **Technology Acceptance Survey** regarding your opinions on the Olera website in relation to your **experiences and needs as a caregiver of a person living with dementia.**

Q2 What is your **full name**?

Q3 What is the **best email** we can keep on file for you?

The following information is to collect your demographic information for the CARES study. 
 
**Your privacy is our priority** with top data protection policies! No identifying information will be shared.

Q4 **What is your age today?**

Q5 **What is your gender?**

1. Female
2. Male
3. Other (specify)

Q6 What is your **relationship status**?

1. Married
2. Widowed
3. Divorced
4. Separated
5. Never married
6. Other (please specify)

Q7 **How many people are currently in your household?**

Q8 What is the highest level of **school you have completed**?

1. Less than high school degree
2. High school degree or equivalent (e.g., GED)
3. Some college, but no degree
4. Associate degree
5. Bachelor degree
6. Graduate degree

Q9 Which of the following categories best describes your **current employment status**?

1. Employed part-time
2. Employed full-time
3. Not employed, looking for work
4. Not employed, NOT looking for work
5. Retired
6. Disabled, unable to work

Q10 How much total combined income did all members of your **household earn in 2022**?

1. Below $50,000
2. $50,000-$100,000
3. Above $100,000

Q11 Please select **the highest** **functional level of difficulty best describes your care recipient from the options below:**

1. No difficulties, either subjectively or objectively.
2. Complains of forgetting location of objects. Subjective word finding difficulties.
3. Decreased job function evident to co-workers; difficulty in traveling to new locations. Decreased organizational capacity.
4. Decreased ability to perform complex tasks (e.g., planning dinner for guests), handling personal finances (forgetting to pay bills), difficulty marketing, etc.
5. Requires assistance in choosing proper clothing to wear for day, season, occasion.
6. 6a- Difficulty putting clothing on properly without assistance.
7. 6b- Unable to bathe properly; e.g., difficulty adjusting bath water temperature) occasionally or more frequently over the past weeks.
8. 6c- Inability to handle mechanics of toileting (e.g., forgets to flush the toilet, does not wipe properly or properly dispose of toilet tissue) occasionally or more frequently over the past weeks.
9. 6d- Urinary incontinence, occasional or more frequent.
10. 6e- Fecal Incontinence, (occasional or more frequently over the past week).
11. 7a- Ability to speak limited to approximately a half dozen different words or fewer, in the course of an average day or in the course of an intensive interview.
12. 7b- Speech ability limited to the use of a single intelligible word in an average day or in the course of an interview (the person may repeat the word over and over.
13. 7c- Ambulatory ability lost (cannot walk without personal assistance).
14. 7d- Ability to sit up without assistance lost (e.g., the individual will fall over if there are no lateral rests [arms] on the chair).
15. 7e- Loss of the ability to smile.

Q12 On a scale of 1 to 5, **how would you rate your current technological expertise?**(1 = Poor; 5 = Excellent)

Q13 On a scale of 1 to 5, how would you rate **your current digital and media literacy?**(1 = Poor; 5 = Excellent)

Q14 Can you **write a personal letter or short message in English**?

1. Not at all
2. With difficulty
3. Easily

Q15 Can you confidently **fill medical forms in the correct manner**?

1. Not at all
2. With difficulty
3. Easily

Q16 Can you accurately **follow the instructions from medical professionals** regarding medication, treatment plans, and post-care procedures?

1. Not at all
2. With difficulty
3. Easily

Q17 Can you **read and understand written health information, including educational materials, brochures, and digital content?**

1. Not at all
2. With difficulty
3. Easily

Q18 Can you **read and understand all the information on prescription labels and healthcare documents**?

1. Not at all
2. With difficulty
3. Easily

Q19 Can you **understand what healthcare providers are explaining or recommending**, including diagnoses, treatment options, and follow-up care?

1. Not at all
2. With difficulty
3. Easily

Q20 When a **new technology** (hardware, software, or web application) becomes available, **how quickly do you tend to adopt it**?

1. I never adopt it
2. After most of my peers
3. When it becomes popular
4. Before most of my peers
5. I'm one of the first to try it

Q21 How often did you **interact with the Olera website**?

1. Daily
2. 4-6 times a week
3. 2-3 times a week
4. Once a week
5. Never

Q22 What were the **total minutes**you spent interacting with the Olera website over the **past four weeks**?

Please rate the **Olera website** based on the criteria outlined in each question.

Q23 The quality of the content was **good**.

1. Strongly agree
2. Agree
3. Somewhat agree
4. Neutral
5. Disagree
6. Strongly disagree

Q24 The quantity of the content was **good**.

1. Strongly agree
2. Agree
3. Somewhat agree
4. Neutral
5. Disagree
6. Strongly disagree

Q25 The content was **relevant** to my caregiving concerns/ needs.

1. Strongly agree
2. Agree
3. Somewhat agree
4. Neutral
5. Disagree
6. Strongly disagree

Q26 The **arrangement and size of buttons and content** on the screen was **functional.**

1. Strongly agree
2. Agree
3. Somewhat agree
4. Neutral
5. Disagree
6. Strongly disagree

Q27 The application was **useful in finding the resources/ services** I needed for caregiving.

1. Strongly agree
2. Agree
3. Somewhat agree
4. Neutral
5. Disagree
6. Strongly disagree

Q28 The application was helpful in finding relevant caregiving information and services **more quickly.**

1. Strongly agree
2. Agree
3. Somewhat agree
4. Neutral
5. Disagree
6. Strongly disagree

Q29 I would consider this website **important** in finding caregiving information and services.

1. Strongly agree
2. Agree
3. Somewhat agree
4. Neutral
5. Disagree
6. Strongly disagree

Q30 This application helped me find **MORE** educational resources about dementia and/or caregiving services.

1. Strongly agree
2. Agree
3. Somewhat agree
4. Neutral
5. Disagree
6. Strongly disagree

Q31 This application helped **filter content** so that I could find topics of interest **quicker.**

1. Strongly agree
2. Agree
3. Somewhat agree
4. Neutral
5. Disagree
6. Strongly disagree

Q32 **Overall**, this application was effective in helping me address my caregiving concerns.

1. Strongly agree
2. Agree
3. Somewhat agree
4. Neutral
5. Disagree
6. Strongly disagree

Q33 The Olera platform made it **easier to find** caregiving resources/ services.

1. Strongly agree
2. Agree
3. Somewhat agree
4. Neutral
5. Disagree
6. Strongly disagree

Q34 It would be **more difficult** to find relevant resources and services **without the Olera platform.**

1. Strongly agree
2. Agree
3. Somewhat agree
4. Neutral
5. Disagree
6. Strongly disagree

Q35 The Olera platform **saved me time** in finding relevant information.

1. Strongly agree
2. Agree
3. Somewhat agree
4. Neutral
5. Disagree
6. Strongly disagree

Q36 I accomplished **more work and/or tasks** with the Olera platform.

1. Strongly agree
2. Agree
3. Somewhat agree
4. Neutral
5. Somewhat disagree
6. Disagree
7. Strongly disagree

Q37 What are your **suggestions for improvement on the functionality** (design, layout, font, etc.) of the Olera platform?

Please rate the following statements regarding your experiences with the **Olera website**.

Q38 The Olera platform was **confusing or difficult to navigate.**

1. Strongly agree
2. Agree
3. Somewhat Agree
4. Neutral
5. Somewhat disagree
6. Disagree
7. Strongly disagree

Q39 I often encountered **errors** while using the Olera platform (i.e. pages not loading or links leading to incorrect pages).

1. Strongly agree
2. Agree
3. Somewhat Agree
4. Neutral
5. Somewhat disagree
6. Disagree
7. Strongly disagree

Q40 It was **frustrating** to use the Olera platform.

1. Strongly agree
2. Agree
3. Somewhat Agree
4. Neutral
5. Somewhat disagree
6. Disagree
7. Strongly disagree

Q41 The **walkthrough** of the website was useful.

1. Strongly agree
2. Agree
3. Somewhat Agree
4. Neutral
5. Somewhat disagree
6. Disagree
7. Strongly disagree

Q42 It was **mentally exhausting** **or draining** to navigate the website and application.

1. Strongly agree
2. Agree
3. Somewhat Agree
4. Neutral
5. Somewhat disagree
6. Disagree
7. Strongly disagree

Q43 The Olera website was **rigid and inflexible.**

1. Strongly agree
2. Agree
3. Somewhat Agree
4. Neutral
5. Somewhat disagree
6. Disagree
7. Strongly disagree

Q44 The website's features were **easy to control and customize.**

1. Strongly agree
2. Agree
3. Somewhat Agree
4. Neutral
5. Somewhat disagree
6. Disagree
7. Strongly disagree

Q45 The content was **easily understandable.**

1. Strongly agree
2. Agree
3. Somewhat Agree
4. Neutral
5. Somewhat disagree
6. Disagree
7. Strongly disagree

Q46 I found the Olera platform **inconvenient and/or unorganized.**

1. Strongly agree
2. Agree
3. Somewhat Agree
4. Neutral
5. Somewhat disagree
6. Disagree
7. Strongly disagree

Q47 It was easy to **remember how to use** the website's features.

1. Strongly agree
2. Agree
3. Somewhat Agree
4. Neutral
5. Somewhat disagree
6. Disagree
7. Strongly disagree

Q48 The Olera platform provides **adequate guidance/ information** for relevant caregiving resources/ services.

1. Strongly agree
2. Agree
3. Somewhat Agree
4. Neutral
5. Somewhat disagree
6. Disagree
7. Strongly disagree

Q49 The platform was **easy to use.**

1. Strongly agree
2. Agree
3. Somewhat Agree
4. Neutral
5. Somewhat disagree
6. Disagree
7. Strongly disagree

Q50 It was **easy to go to third party websites** **and return** to the Olera platform.

1. Strongly agree
2. Agree
3. Somewhat Agree
4. Neutral
5. Somewhat disagree
6. Disagree
7. Strongly disagree

Q51 It was **easy to learn how to use** the Olera platform.

1. Strongly agree
2. Agree
3. Somewhat Agree
4. Neutral
5. Somewhat disagree
6. Disagree
7. Strongly disagree

Q52 I used **little to no effort** to become skillful in navigating the Olera platform.

1. Strongly agree
2. Agree
3. Somewhat Agree
4. Neutral
5. Somewhat disagree
6. Disagree
7. Strongly disagree

Q53 Did you encounter any **unexpected features or experiences** while using the website and application? If so, please describe in 1-3 sentences.

Q54 What is your **overall feedback on the relevancy or usefulness of the recommended caregiving services or educational articles**?

An **online forum** is a **discussion space/ online board** where individuals can post **questions answers, or discussions** on a specific topic. The follow questions are about the newly developed online forum by Olera, the Caregiver Community Forum.

Q55 Have you **ever posted or used a forum on other websites before, such as Reddit, Quora, Yahoo, Facebook**? If so, which ones? (select all that apply)

1. Reddit
2. Quora
3. Yahoo
4. Facebook
5. Discord
6. Other
7. I have never posted or used another website forum

Q55 Have you **ever used Olera Caregiver Forum**? (select all that apply)

1. I have posted content myself
2. I have responded to other's content/ posts
3. I have browsed for information
4. I have not used it yet

Q56 What would you like to see **improved or different** about the Olera Caregiver Community Forum?

Q57 What would make you **more likely to post or engage with other caregivers**on our Olera Caregiver Community Forum?

Q58 How do you like to receive information on caregiving?

1. Email
2. Text
3. Live video calls
4. Live events (conferences, lectures, etc)
5. Church support groups
6. Books
7. Forums
8. Online Blogs/Articles
9. YouTube
10. Social Media Posts or Groups (Facebook, etc.)
11. Other online videos
12. Other (Please specify

**The term ‘artificial intelligence’ means a machine-based system that can, for a given set of human-defined objectives, make predictions, recommendations or decisions influencing real or virtual environments.**

Q59 Would you feel confident in AI-assisted caregiving support, such as finding caregiving education and services for you?

1. Yes
2. No

Q60 What would make you more confident in AI-assisted caregiving support services?

Q61 What was the hardest part about your caregiver journey?

Q62 What is your biggest barrier to obtaining the care you need for your loved one with dementia?

1. Information
2. Senior Living
3. Financial
4. Legal
5. Medical
6. Time
7. Mental Strain
8. Emotional Strain
9. Other (please specify)

Q63 Could you tell us more about how your biggest barrier to obtaining you need for your loved one with dementia?

Q64 Is there anything else you want to tell us about caregiving for our research study to better understand the challenges modern-day caregivers face?
