# Supplementary material for: Exploring Informal Caregivers’ Perception of the Olera Digital Caregiving Assistance Platform for Dementia Care: Mixed Methods Evaluation Study
Source: JMIR Form Res. 2026 Jul 3;10:e92967. doi: 10.2196/92967 (PMC13331331; doi:10.2196/92967)
Supplement: Multimedia Appendix 6 [file formative-v10-e92967-s006.docx]

**Extended table of perceived acceptance of technology by all participant characteristics collected (n=65).**

| **Variables** | | **N (%) or Mean (SD)** | **TAS score (SD)** | **Test statistics** |
| --- | --- | --- | --- | --- |
| **Demographics and caregiving characteristics** | | | | |
| **Age (in years)** | | 59.9 (9.8) | 5.83 (0.85) | Linear regression  β=−.007, p=.546 |
| **Gender** | |  |  | N/A |
| Female | | 61 (93.8%) | 5.83 (0.84) |  |
| Male | | 3 (4.7%) | 5.63 (1.36) |  |
| Did not respond | | 1 (1.5%) |  |  |
| **Race or ethnicity** | |  |  | N/A |
| White or Caucasian | | 45 (69.2%) | 5.79 (0.82) |  |
| Black or African American | | 9 (13.8%) | 6.06 (0.84) |  |
| Hispanic or Latino | | 6 (9.2%) | 6.37 (0.61) |  |
| Others (Asian, Native American or Alaskan Native, Multiracial) | | 5 (7.7%) | 5.13 (1.07) |  |
| **Relationship status** | |  |  | N/A |
| Married | | 37 (56.9%) | 5.94 (0.89) |  |
| Never married | | 12 (18.5%) | 5.26 (0.72) |  |
| Widowed, divorced, or separated | | 14 (21.5%) | 5.95 (0.71) |  |
| Other (significant other) | | 1 (1.5%) | 6.52 (0.00) |  |
| Did not respond | | 1 (1.5%) |  |  |
| **Household size** | | 2.5 (1.0) | 5.83 (0.85) | Linear regression  β=−.016, p=0.882 |
| **Education** | |  |  | N/A |
| Graduate degree | | 16 (24.6%) | 5.60 (0.94) |  |
| Bachelor’s degree | | 21 (32.3%) | 5.86 (0.79) |  |
| Associate degree | | 9 (13.8%) | 6.10 (1.12) |  |
| Some college, but no degree | | 14 (21.5%) | 5.83 (0.68) |  |
| High school degree or equivalent | | 4 (6.2%) | 5.90 (0.93) |  |
| Did not respond | | 1 (1.5%) |  |  |
| **Employment status** | |  |  | N/A |
| Retired | | 25 (38.5%) | 5.83 (0.82) |  |
| Full-time employed | | 14 (21.5%) | 6.08 (0.76) |  |
| Part-time employed | | 11 (16.9%) | 5.51 (0.98) |  |
| Not employed, not looking for work | | 11 (16.9%) | 6.10 (0.54) |  |
| Not employed, looking for work | | 2 (3.1%) | 4.34 (1.51) |  |
| Disabled, unable to work | | 1 (1.5%) | 5.48 (0.00) |  |
| Did not respond | | 1 (1.5%) |  |  |
| **Total household income** | |  |  | N/A |
| Above $100,000 | | 9 (13.8%) | 6.01 (0.49) |  |
| $50,000–$100,000 | | 39 (60.0%) | 5.82 (0.94) |  |
| Below $50,000 | | 16 (24.6%) | 5.73 (0.81) |  |
| Did not respond | | 1 (1.5%) |  |  |
| **Relationship with care recipients** | | |  | N/A |
| Adult child | | 42 (64.6%) | 5.85 (0.77) |  |
| Family member | | 6 (9.2%) | 6.16 (0.83) |  |
| Legal guardian | | 1 (1.5%) | 3.28 (0.00) |  |
| Spouse or partner | | 16 (24.6%) | 5.83 (0.85) |  |
| **Digital literacy and technology use** | | | | |
| **Technology proficiency** |  | |  | N/A |
| 1- poor | 1 (1.5%) | | 5.00 (0.00) |  |
| 2- fair | 2 (3.1%) | | 5.00 (0.68) |  |
| 3- good | 19 (29.2%) | | 5.91 (0.75) |  |
| 4- very good | 21 (32.3%) | | 5.69 (0.89) |  |
| 5- excellent | 22(33.9%) | | 6.00 (0.88) |  |
| **Digital/media literacy** |  | |  | N/A |
| 1- poor | 2 (3.1%) | | 5.24 (0.34) |  |
| 2- fair | 1 (1.5%) | | 4.51 (0.00) |  |
| 3- good | 15 (23.1%) | | 5.71 (0.64) |  |
| 4- very good | 28 (43.1%) | | 5.83 (0.92) |  |
| 5- excellent | 19 (29.2%) | | 6.05 (0.88) |  |
| **Adoption of new technology** |  | |  | ANOVA F(3,61)= 1.58,  p = .203 |
| When it becomes popular | 24 (36.9%) | | 5.66 (1.07) |  |
| After most peers | 17 (26.1%) | | 5.67 (0.67) |  |
| Before most peers | 15 (23.1%) | | 6.19 (0.61) |  |
| One of the first to try | 9 (13.9%) | | 6.00 (0.71) |  |
| **Olera platform interaction frequency** |  | |  | ANOVA F(3,61)= 7.88,  p < .001 |
| Daily | 10 (15.4%) | | 6.55 (0.44) |  |
| 4–6 times/week | 18 (27.7%) | | 6.05 (0.70) |  |
| 2–3 times/week | 20 (30.8%) | | 5.82 (0.62) |  |
| Once/week | 17 (26.1%) | | 5.20 (0.99) |  |
| **Olera platform use (in minutes over past 4 weeks)** | 187.0 (167.0) | | 5.83 (0.85) | Linear regression  β=.002, p=.013 |
| **Health literacy items** | | | |  |
| **Can write a personal letter in English** | | |  | N/A |
| Easily | | 65 (100.0%) | 5.83 (0.85) |  |
| With difficulty | | 0 (0.0%) | N/A |  |
| **Can confidently fill medical forms** | |  |  | N/A |
| Easily | | 64 (98.5%) | 5.84 (0.85) |  |
| With difficulty | | 1 (1.5%) | 5.48 (N/A) |  |
| **Can accurately follow medical instructions** | | |  | N/A |
| Easily | | 65 (100.0%) | 5.83 (0.85) |  |
| With difficulty | | 0 (0.0%) | N/A |  |
| **Can read/understand written health info** | | |  | N/A |
| Easily | | 63 (96.9%) | 5.83 (0.86) |  |
| With difficulty | | 2 (3.1%) | 5.88 (0.56) |  |
| **Can read/understand prescription labels** | | |  | N/A |
| Easily | | 62 (95.4%) | 5.82 (0.87) |  |
| With difficulty | | 3 (4.6%) | 6.18 (0.09) |  |
| **Can understand provider explanations** | | |  | N/A |
| Easily | | 61 (95.3%) | 5.82 (0.87) |  |
| With difficulty | | 3 (4.7%) | 5.89 (0.40) |  |

Note: This appendix presents descriptive subgroup summaries and selected exploratory analyses of overall TAS scores. Categories with sparse cell sizes were retained for descriptive completeness only and were not interpreted inferentially. These analyses were exploratory and should be interpreted cautiously.

N/A: Not tested because one or more categories had sparse cell sizes (≤5 participants), making inferential comparisons unstable.
