# Supplementary material for: Exploring Informal Caregivers’ Perception of the Olera Digital Caregiving Assistance Platform for Dementia Care: Mixed Methods Evaluation Study
Source: JMIR Form Res. 2026 Jul 3;10:e92967. doi: 10.2196/92967 (PMC13331331; doi:10.2196/92967)
Supplement: Multimedia Appendix 7 [file formative-v10-e92967-s007.docx]

**Comparison of characteristics between participants who completed the study and those lost to follow up.**

|  | **A**  **Participants who completed study (n=65)** | **B**  **Participants lost to follow up (n=88)** |  |
| --- | --- | --- | --- |
|  | **n (%) or Mean** | **n (%) or Mean** |  |
| **Care recipient relationship** | | |  |
| Adult child | 42 (64.6%) | 40 (45.5%) | Fisher’s exact test  *p*= 0.097 |
| Family member | 6 (9.2%) | 15 (17%) |  |
| Legal guardian | 1 (1.5%) | 1 (1.1%) |  |
| Spouse or partner | 16 (24.6%) | 31 (35.2%) |  |
| Did not respond | 0 (0%) | 1 (1.1%) |  |
| **Race and ethnicity** | | | |
| White or Caucasian | 45 (69.2%) | 60 (68.2%) | Chi-square test *p*= 0.498 |
| Black or African American | 9 (13.8%) | 11 (12.5%) |  |
| Hispanic or Latin American | 6 (9.2%) | 11 (12.5%) |  |
| Others (Asian, Native American or Alaskan Native, Multiracial) | 5 (7.7%) | 6 (6.7%) |  |
| **Currently looking for caregiving services** | | | |
| Yes | 41 (63.1%) | 65 (73.9%) | Fisher’s exact test *p=* *0.154* |
| No | 24 (36.9%) | 22 (25%) |  |
| Did not respond | 0 (0.0%) | 1 (1.1%) |  |
| **Age of caregiver (in years)** | 59.9 | 61.9 | t-test *p* *= 0.195* |
| **Age of care recipient (in years)** | 79.5 | 77.4 | t-test *p* *=0.151* |
| **Hours caregiving per week** | 83.6 | 98.8 | t-test *p =0.428* |
| **Years caregiving** | 5.6 | 5.9 | t-test *p =0.788* |
