# Supplementary material for: Exploring Informal Caregivers’ Perception of the Olera Digital Caregiving Assistance Platform for Dementia Care: Mixed Methods Evaluation Study
Source: JMIR Form Res. 2026 Jul 3;10:e92967. doi: 10.2196/92967 (PMC13331331; doi:10.2196/92967)
Supplement: Multimedia Appendix 8 [file formative-v10-e92967-s008.docx]

**Mean and standard deviation (SD) of Technology Acceptance Survey items (n=65).**

| **TAS Item** | **Mean (SD)** |
| --- | --- |
| 1. The quality of the content was good. | 6.25 (1.02) |
| 1. The quantity of the content was good. | 6.15 (0.85) |
| 3. The content was relevant to my caregiving concerns/ needs. | 5.97 (1.41) |
| 4. The arrangement and size of buttons and content on the screen was functional. | 6.37 (0.72) |
| 5. The application was useful in finding the resources/ services I needed for caregiving. | 5.78 (1.52) |
| 5. The application was helpful in finding relevant caregiving information and services more quickly. | 5.88 (1.28) |
| 6. I would consider this website important in finding caregiving information and services. | 6.06 (1.07) |
| 7. This application helped me find more educational resources about dementia and/or caregiving services. | 5.83 (1.29) |
| 8. This application helped filter content so that I could find topics of interest quicker. | 5.72 (1.28) |
| 9. Overall, this application was effective in helping me address my caregiving concerns. | 5.85 (1.38) |
| 10. The Olera platform made it easier to find caregiving resources/ services. | 5.80 (1.46) |
| 11. It would be more difficult to find relevant resources and services without the Olera platform. | 5.43 (1.50) |
| 12. The Olera platform saved me time in finding relevant information. | 5.66 (1.36) |
| 13. I accomplished more work and/or tasks with the Olera platform. | 5.31 (1.38) |
| 14. The Olera platform was confusing or difficult to navigate. | 5.75 (1.35) |
| 15. I often encountered errors while using the Olera platform (i.e. pages not loading or links leading to incorrect pages). | 5.43 (1.54) |
| 16. It was frustrating to use the Olera platform. | 6.00 (1.24) |
| 17. The walkthrough of the website was useful. | 5.60 (1.40) |
| 18. It was mentally exhausting or draining to navigate the website and application. | 5.97 (1.41) |
| 19. The Olera website was rigid and inflexible. | 5.84 (1.29) |
| 20. The website's features were easy to control and customize. | 5.49 (1.13) |
| 21. The content was easily understandable. | 6.26 (0.78) |
| 22. I found the Olera platform inconvenient and/or unorganized. | 6.02 (1.30) |
| 23. It was easy to remember how to use the website's features. | 5.91 (1.10) |
| 24. The Olera platform provides adequate guidance/ information for relevant caregiving resources/ services. | 5.58 (1.46) |
| 25. The platform was easy to use. | 6.17 (0.91) |
| 26. It was easy to go to third party websites and return to the Olera platform. | 5.52 (1.23) |
| 27. It was easy to learn how to use the Olera platform. | 5.91 (1.11) |
| 28. I used little to no effort to become skillful in navigating the Olera platform. | 5.68 (1.26) |
| **Overall TAS score** | **5.83 (0.85**) |
