# Supplementary figures and images for: Exploring Informal Caregivers’ Perception of the Olera Digital Caregiving Assistance Platform for Dementia Care: Mixed Methods Evaluation Study
Source: JMIR Form Res. 2026 Jul 3;10:e92967. doi: 10.2196/92967 (PMC13331331; doi:10.2196/92967)

**Technology Acceptance Survey- Likert Responses (100% Stacked)**


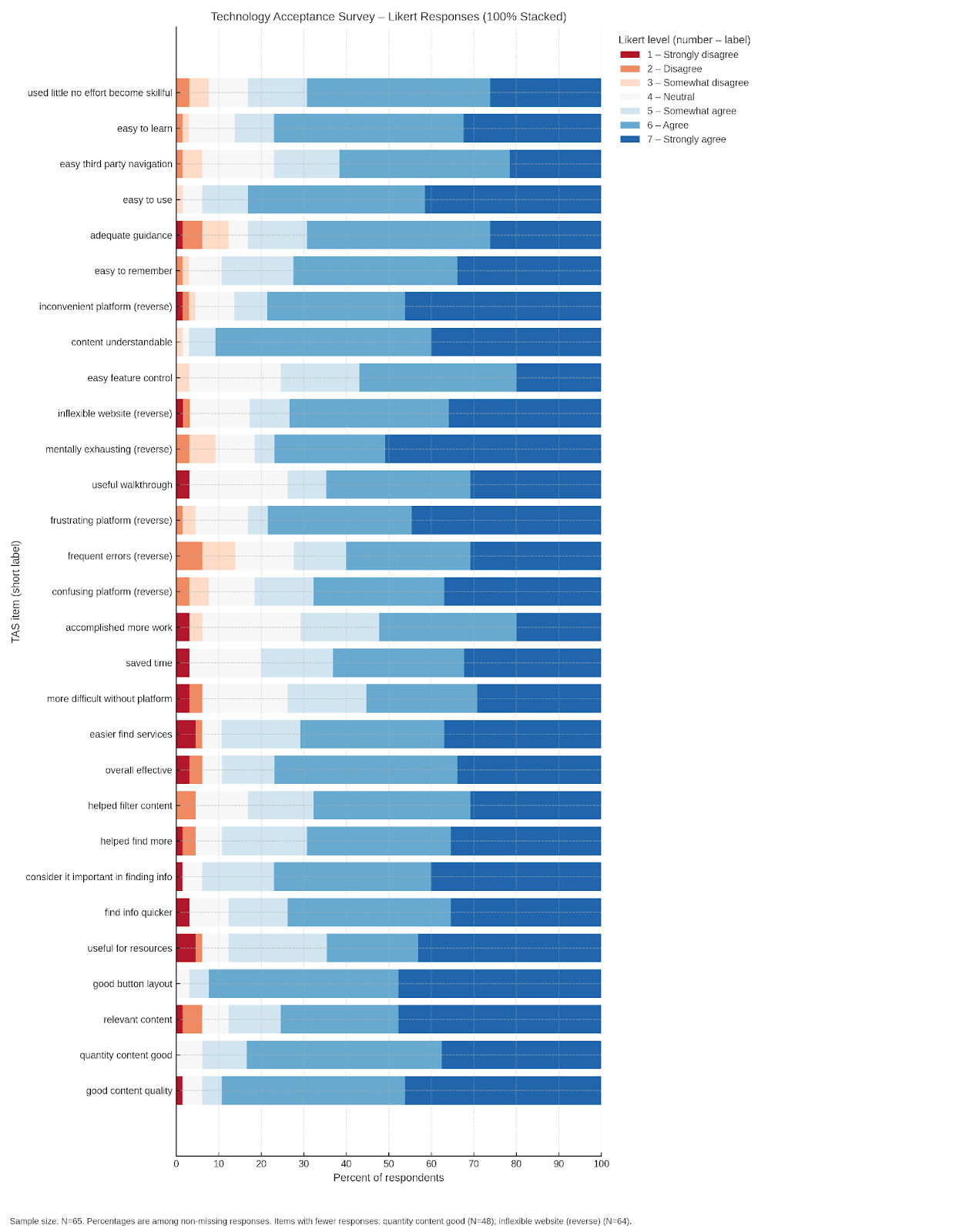

Supplement: Multimedia Appendix 9 [file formative-v10-e92967-s009.docx]
